# A phase-separated CO_2_-fixing pyrenoid proteome determined by TurboID in *Chlamydomonas reinhardtii*

**DOI:** 10.1093/plcell/koad131

**Published:** 2023-05-17

**Authors:** Chun Sing Lau, Adam Dowle, Gavin H Thomas, Philipp Girr, Luke C M Mackinder

**Affiliations:** Centre for Novel Agricultural Products, Department of Biology, University of York, York YO10 5DD, UK; Department of Biology, University of York, York YO10 5DD, UK; Department of Biology, University of York, York YO10 5DD, UK; Centre for Novel Agricultural Products, Department of Biology, University of York, York YO10 5DD, UK; Centre for Novel Agricultural Products, Department of Biology, University of York, York YO10 5DD, UK

## Abstract

Phase separation underpins many biologically important cellular events such as RNA metabolism, signaling, and CO_2_ fixation. However, determining the composition of a phase-separated organelle is often challenging due to its sensitivity to environmental conditions, which limits the application of traditional proteomic techniques like organellar purification or affinity purification mass spectrometry to understand their composition. In *Chlamydomonas reinhardtii*, Rubisco is condensed into a crucial phase-separated organelle called the pyrenoid that improves photosynthetic performance by supplying Rubisco with elevated concentrations of CO_2_. Here, we developed a TurboID-based proximity labeling technique in which proximal proteins in *Chlamydomonas* chloroplasts are labeled by biotin radicals generated from the TurboID-tagged protein. By fusing 2 core pyrenoid components with the TurboID tag, we generated a high-confidence pyrenoid proxiome that contains most known pyrenoid proteins, in addition to new pyrenoid candidates. Fluorescence protein tagging of 7 previously uncharacterized TurboID-identified proteins showed that 6 localized to a range of subpyrenoid regions. The resulting proxiome also suggests new secondary functions for the pyrenoid in RNA-associated processes and redox-sensitive iron–sulfur cluster metabolism. This developed pipeline can be used to investigate a broad range of biological processes in *Chlamydomonas*, especially at a temporally resolved suborganellar resolution.

## Teaser

A high-confidence proteome of the CO_2_-fixing algal pyrenoid is identified by proximity labeling.

## Introduction

Nearly all algae contain a microcompartment in their chloroplast called the pyrenoid, which is estimated to be responsible for ∼30% of global CO_2_ fixation (Mackinder et al. 2016). The pyrenoid of the model green alga *Chlamydomonas* (*Chlamydomonas reinhardtii*) is a 1- to 2-*µ*m biomolecular condensate of the principal CO_2_-fixing enzyme Rubisco. It is formed through liquid–liquid phase separation (LLPS) of Rubisco mediated by ESSENTIAL PYRENOID COMPONENT 1 (EPYC1), which harbors 5 evenly spaced Rubisco-binding motifs (RBMs) interspaced by disordered sequences (Mackinder et al. 2016; Freeman Rosenzweig et al. 2017; Wunder et al. 2018; He et al. 2020). The deletion of *EPYC1*, or the reciprocal binding site of EPYC1 on Rubisco, abolishes pyrenoid formation. Correct pyrenoid assembly is essential for a functional CO_2_-concentrating mechanism (CCM) (Mackinder et al. 2016) that works to saturate Rubisco with CO_2_ to minimize energetically costly photorespiration, thereby improving photosynthetic efficiency (Wang et al. 2015; Fei et al. 2022). In the face of growing food security issues, the engineering of a pyrenoid-based CCM into major C_3_ crop plants such as rice (*Oryza sativa*), soybean (*Glycine max*), and wheat (*Triticum aestivum*) is regarded as a promising strategy for yield improvement, with prospects of increasing food production by up to 60% (Ray et al. 2013; Long et al. 2019). Recent work reconstituted a proto-pyrenoid in the land plant *Arabidopsis* (*Arabidopsis thaliana*) (Atkinson et al. 2020). However, additional structural components, such as those needed to traverse thylakoid membranes and establish a CO_2_ diffusion barrier will be required for efficient function (Fei et al. 2022). Many of the proteins underpinning these additional structural requirements are unknown, making a deep understanding of the structural organization and molecular function of the pyrenoid critical.

Previous pyrenoid proteomes have been achieved via organelle purification (Mackinder et al. 2016; Zhan et al. 2018) and affinity purification followed by mass spectrometry (AP-MS) (Mackinder et al. 2017); however, these methods have limitations. While multiple robust methods, like AP-MS, exist to identify strong protein–protein interactions, the ability to identify weak and transient interactions in vivo is limited. At a larger spatial scale, subcellular fractionation followed by protein purification and MS is prone to cross-contamination (Christopher et al. 2021). Biomolecular condensates, like the pyrenoid, fall into a class of subcellular structures whose proteomes are challenging to accurately determine as they are typically dynamic, involving weak and transient interactions that are highly sensitive to small changes in the surrounding environment, can vary considerably in size, and are not always clearly spatially defined due to the absence of an encapsulating membrane (Hyman et al. 2014; Choi et al. 2020; Barrett et al. 2021). Recently developed proximity labeling methods such as APEX2 and TurboID (Lam et al. 2015; Branon et al. 2018) are particularly poised to determine the transient interactions and proteomes of biomolecular condensates (Bracha et al. 2019). APEX2 and TurboID use an enzyme tag that drives biotinylation of neighboring proteins in vivo. In APEX2, an engineered ascorbate peroxidase converts biotin-phenol to biotin-phenoxyl radicals; with TurboID, an engineered biotin ligase generates biotin-5′-AMP radicals from biotin and ATP (Roux et al. 2012; Branon et al. 2018). These labile radicals spontaneously biotinylate the surface of exposed residues from proteins in close proximity. This reaction gives rise to a localized biotinylation event that is spatially restricted to 10 to 40 nm (Kim et al. 2014, 2016) by the diffusion of the radical from the enzyme tag. This in vivo biotinylation method bypasses the need to purify proteins in their native association, with the high affinity of the biotin tag to streptavidin beads enabling the removal of background contaminants via harsh wash conditions. Proximity labeling thus results in the identification of strong, weak, and transient interactions, in addition to noninteracting proximal proteins. However, since its development, proximity labeling has seen limited application in phase-separated systems (Youn et al. 2018; Zhou and Zou 2021) and has yet to be established in plastids or the alga *Chlamydomonas*.

In this study, we attempted to identify those proteins that were missed by AP-MS and pyrenoid purification by developing a pyrenoid-based proximity labeling methodology. Using TurboID-based proximity labeling, we identify a complementary and robust pyrenoid “proxiome.” Our pyrenoid proxiome contains most previously known pyrenoid proteins and has identified multiple new pyrenoid components that show distinct subpyrenoid localizations, as determined via fluorescence tagging. The ability to identify core proteins involved in pyrenoid phase separation highlights the strength of proximity labeling for investigating biomolecular condensate composition and formation. Furthermore, our method establishes proximity labeling in plastids and the leading model algal system, *Chlamydomonas*.

## Results

### Development of proximity labeling in *Chlamydomonas*

**Figure 1.**
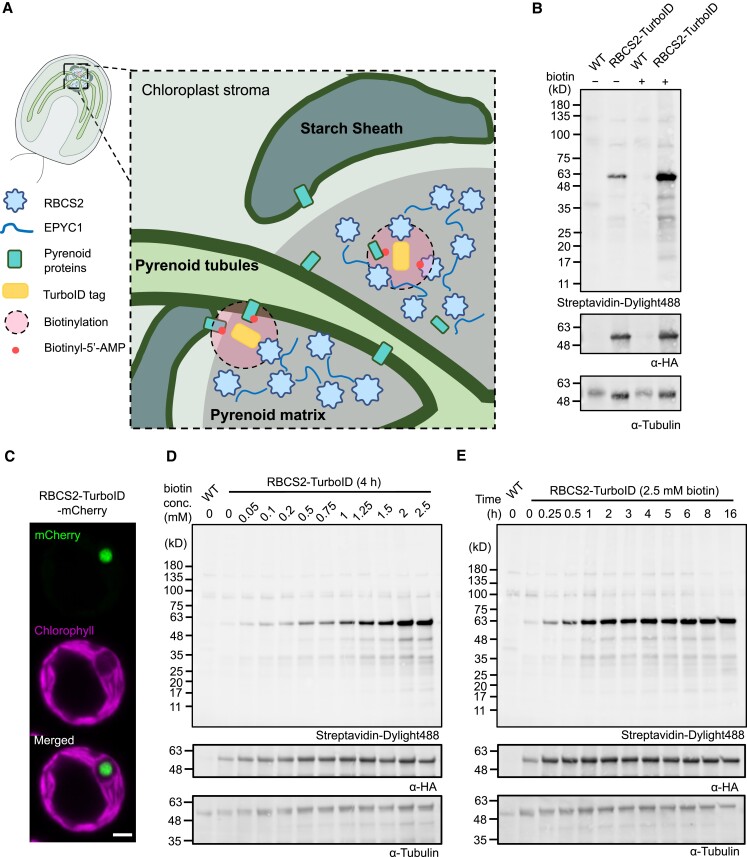
Establishment and optimization of TurboID labeling in the *Chlamydomonas* chloroplast using RBCS2-TurboID lines. **A)** Schematic representation of the *Chlamydomonas* pyrenoid and RBCS2-TurboID. The pyrenoid matrix is surrounded by a starch sheath and traversed by pyrenoid tubules. The RBCS2-TurboID fusion protein is targeted to the pyrenoid matrix; upon addition of the biotin substrate, short-lived biotin radicals (round red dots) diffuse from the TurboID tag and spontaneously biotinylate neighboring pyrenoid proteins. **B)** Biotinylation signals of strains transformed with the *RBCS2-TurboID* construct and the untagged background (WT) were assessed by immunoblotting whole-cell lysate with a streptavidin conjugate. Anti-tubulin was used as a loading control, with anti-HA used to probe for abundance of the fusion protein. **C)** Confocal imaging of RBCS2-TurboID-mCherry. Green and magenta signals represent the mCherry and chlorophyll autofluorescence respectively. Scale bar is 2 *µ*m. **D to E)** RBCS2-TurboID labeling efficiency was determined by labeling cells across a biotin concentration gradient (0 to 2,500 *µ*M) for 4 h **D)** or across a time range (0 to 16 h) with 2.5 mM biotin substrate **E)**.

We set out to establish proximity labeling in the LLPS pyrenoid within the *Chlamydomonas* chloroplast (Fig. 1A). TurboID has been established in *Arabidopsis* (Zhang et al. 2019; Mair and Bergmann 2022) and APEX2 in cyanobacteria (Dahlgren et al. 2021) and diatoms (Turnšek et al. 2021). To determine which approach is best suited for *Chlamydomonas*, we designed constructs to test both APEX2 and TurboID (Supplemental Data Set 1). We designed expression constructs to be compatible with the *Chlamydomonas* modular cloning (MoClo) framework (Crozet et al. 2018) to enable community adoption and compatibility with a broad range of promoters, terminators, and selection markers.

We initially chose the Rubisco small subunit 2 (RBCS2, encoded by Cre02.g120150) as our bait due to (i) the central role of Rubisco in pyrenoid LLPS (Meyer et al. 2012; Wunder et al. 2018); (ii) previous data showing that tagging exogenous RBCS does not affect CCM functionality (Freeman Rosenzweig et al. 2017); and (iii) the availability of known interacting partners for downstream validation (Mackinder et al. 2017; Meyer et al. 2020). We thus fused either the APEX2 or the TurboID tag to the C-terminus of RBCS2 and placed the encoding cassette under the control of the well-established *PSAD* promoter/terminator pair previously used for fluorescence protein tagging of a broad range of pyrenoid components including RBCS2 (Mackinder et al. 2017). We transformed all constructs individually via electroporation into the widely used wild-type (WT) strain CC-4533 (Li et al. 2016, 2019). We screened hygromycin-resistant colonies for genomic insertion of the *RBCS2* fusion construct via PCR and then for protein accumulation by immunoblotting against the C-terminal epitope tag (Supplemental Figs. S1A and S2A). We named the resulting strains harboring each construct RBCS2-APEX2 and RBCS2-TurboID.

We confirmed the correct localization of RBCS2-APEX2 to the pyrenoid by immunofluorescence against the 3xFlag tag at the C-terminus of APEX2 (Supplemental Fig. S1B). To validate the activity of RBCS2-APEX2, we incubated RBCS2-APEX2 strain A2 (Supplemental Fig. S1A) with the biotin-phenol substrate, which showed a subtle yet different biotinylation pattern from that of the untagged WT background, especially when activated with higher H_2_O_2_ concentration (Supplemental Fig. S1C). This observation led us to pursue a preliminary labeling experiment followed by MS of affinity-purified biotinylated proteins. Analysis of these data showed minimal enrichment for Rubisco or known pyrenoid components (Supplemental Fig. S1D). However, when assessing APEX2 peroxidase activity using Amplex Red, we detected higher peroxidase activity in RBCS2-APEX2 than in its untagged counterpart, suggesting that the fusion protein is functional (Supplemental Fig. S1E). We tentatively conclude that biotin-phenol has limited cellular permeability resulting in poor labeling. This poor permeability agrees with previous reports in budding yeast (*Saccharomyces cerevisiae*), where cell wall modification was required to facilitate biotin-phenol uptake (Hwang and Espenshade 2016; Li et al. 2020). The failure of APEX2 to work in *Chlamydomonas* was also reported by Kreis et al. (2022).

By contrast, initial tests of RBCS2-TurboID showed clear increased biotinylation in comparison to WT with the addition of the biotin substrate (Fig. 1B). We observed a pronounced band at ∼50 kD that likely corresponds to either the self-biotinylation of the RBCS2-TurboID fusion protein (55 kD) or the Rubisco large subunit (55 kD) (Fig. 1B). We also observed a weak biotinylation signal in the absence of external biotin addition, indicating that naturally occurring biotin is present in the chloroplast, as suggested by the presence of endogenously biotinylated chloroplast proteins (Li-Beisson et al. 2015).

After demonstrating TurboID activity, we assessed the localization of the fusion protein by generating a RBCS2-TurboID-mCherry fusion. Confocal imaging confirmed its pyrenoid localization, with the mCherry signal forming a single punctum at the canonical pyrenoid position characterized by an absence of chlorophyll fluorescence signal (Fig. 1C). We next optimized the concentration of the biotin substrate and labeling time (Fig. 1, D and E). To this end, we grew cells photoautotrophically with air-level CO_2_ supplementation to induce the CCM, which leads to nearly all Rubisco being condensed into the pyrenoid (Borkhsenious et al. 1998). We then incubated these cells with a range of biotin concentrations (0.1 to 2.5 mM) over different time periods (1 to 16 h). We determined that biotin labeling occurs in a substrate- (Fig. 1D) and time- (Fig. 1E) dependent manner. In contrast to land plants where labeling saturation can be achieved with 50 *µ*M biotin (Mair et al. 2019; Wurzinger et al. 2022), labeling in *Chlamydomonas* appears to saturate at the much higher biotin concentration of 2.5 mM. This result is in line with Kreis et al. (2022) with the use of a 1 mM concentration. To maximize labeling, we performed all later experiments using a final concentration of 2.5 mM biotin. In agreement with previous reports (Mair et al. 2019; Zhang et al. 2019), we similarly observed the rapid activity by TurboID, which allowed labeling to approach saturation after ∼1 h (Fig. 1E).

### RBCS2-TurboID labels Rubisco interactors and pyrenoid proteins

**Figure 2.**
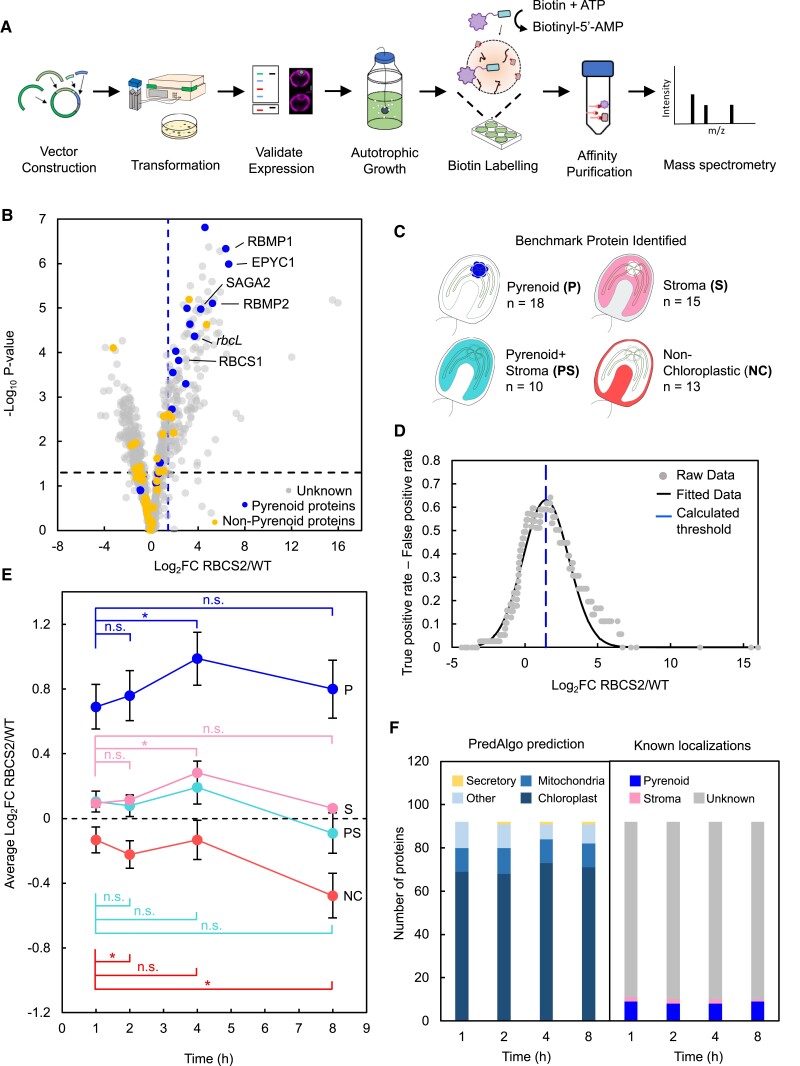
TurboID pipeline development and optimization of labeling time. **A)** Schematic representation of the developed TurboID pipeline. **B)** Volcano plot representing Log_2_ FC between protein abundance in RBCS2-TurboID and WT. Proteins are colored according to their localization: unknown (gray), pyrenoid proteins (blue), and other localizations including chloroplast stroma, pyrenoid + stroma, and nonchloroplastic (yellow). The Log_2_ FC threshold (dashed blue line) was calculated via the ROC analysis where only pyrenoid proteins are considered true positives. −Log_10_*P*-value was used to represent statistical significance from the 1-way ANOVA test carried out on the difference in abundance between RBCS2-TurboID and WT. *P*-value of <0.05 was used as a threshold. **C)** Benchmark proteins detected from the RBCS2-TurboID sample; a complete list of benchmark proteins used is given in Supplemental Data Set 3. **D)** Trade-off between the true-positive rate and false-positive rates plotted against the Log_2_ FC value. A Gaussian function was fitted to the experimental data to determine a maximum, which was used as the enrichment threshold used in **B)**. **E)** Log_2_ FC of RBCS2-TurboID according to localization category in **C)** calculated at each labeling time point. Statistical significance was tested between time points within each class of benchmark proteins by a 2-way repeated measures ANOVA. *: *P* < 0.01; n.s.: nonsignificant comparison (Supplemental Data Set 8). **F)** PredAlgo-predicted localization and benchmark protein categories of the top 10% enriched proteins from RBCS2-TurboID at each labeling time point.

We established a pipeline for streptavidin affinity purification and protein identification by liquid chromatography–tandem MS (LC-MS/MS) (Fig. 2A; Materials and methods). Due to the relatively high levels of background biotinylation, we set out to further optimize labeling time in a pilot experiment. Accordingly, we incubated RBCS2-TurboID and the untagged WT strains with 2.5 mM biotin across a range of durations (1, 2, 4, and 8 h). We then subjected proteins extracted from the labeled cells to affinity purification with streptavidin magnetic beads. We detected a total of 918 proteins by LC-MS/MS across all samples. Initial results showed a strong enrichment for core pyrenoid localized proteins, including RBCS1, RbcL, EPYC1, STARCH GRANULES ABNORMAL 2 (SAGA2), RUBISCO-BINDING MEMBRANE PROTEIN 1 (RBMP1), and RBMP2 when compared to WT cells not expressing *RBCS2-TurboID* (Fig. 2B and Supplemental Data Set 2). Using the detected proteins, we manually curated 4 benchmark protein sets with known localizations from the literature, namely, pyrenoid-specific proteins (P; 18 proteins), proteins found in the pyrenoid and the stroma (PS; 10 proteins), proteins found in the stroma but excluded from the pyrenoid (S; 15 proteins), and nonchloroplast proteins (NC; 13 proteins) (Fig. 2C and Supplemental Data Set 3). We used these benchmark proteins to calculate the enrichment threshold used to assess significant pyrenoid enrichment by applying a receiver–operator characteristic (ROC) analysis (Branon et al. 2018; Fig. 2D). For the ROC analysis, we adopted a stringent threshold by considering true positive proteins as exclusively pyrenoid-localized (P) proteins. It should be noted that a portion of the pyrenoid-localized proteins used for ROC analysis does not partition within the LLPS pyrenoid matrix but localizes to the starch plate or the pyrenoid tubules. However, we reasoned that their close association to the pyrenoid would still support their labeling by RBCS2-TurboID.

We then investigated protein labeling by RBCS2-TurboID at each time point for the different benchmark sets (Fig. 2E). We established that pyrenoid-localized proteins (P) consistently show the highest labeling across all time points, with both Pyrenoid proteins and Stromal proteins exhibiting a statistically significant increase in labeling from 1 to 4 h, while nonchloroplastic proteins remained stable. Interestingly, all benchmark proteins appear to decrease in labeling at the 8-h time point. This decrease is due to an increase in biotinylated protein abundance in untagged WT, rather than lower labeling by RBCS2-TurboID (Supplemental Data Set 2). While further testing on finer time points will be required to establish the true saturation point in biotin labeling, our data suggest that protein labeling by RBCS2-TurboID begins to approach saturation around 4 h. When we compared the top 10% of enriched proteins across the 4 time points, we detected consistent agreement with their predicted cellular localization, consistent enrichment for pyrenoid-localized proteins (Fig. 2F) and a >72% overlap in protein identity (Supplemental Data Set 2).

Collectively, most pyrenoid-localized proteins can be enriched within the first hour; however, increasing incubation time leads to increased biotinylation. Excluding the 8-h time point due to its increased background abundance, the largest differences between pyrenoid proteins, pyrenoid-excluded stromal proteins and nonchloroplast proteins occur at 4 h. We thus opted for 4-h incubations for later experiments. We hypothesize that the rapid labeling dynamics of pyrenoid proteins within the first hour and the slower increase in labeling of stromal proteins can be explained by the LLPS properties of the pyrenoid where Rubisco is present in both the condensed phase (pyrenoid) and dilute phase (stroma). The high concentration of Rubisco in the condensed phase enables rapid labeling of proximal pyrenoid proteins. However, as Rubisco is also in the dilute phase at a much lower concentration, stromal proteins are biotinylated at a slower rate. This idea is further supported by experimental studies that show, under similar growth conditions used for our experiments, that ∼90% of Rubisco is in the pyrenoid with the rest in the stroma (Borkhsenious et al. 1998; Mackinder et al. 2016).

### Stromal-TurboID controls enable a refined pyrenoid proteome

**Figure 3.**
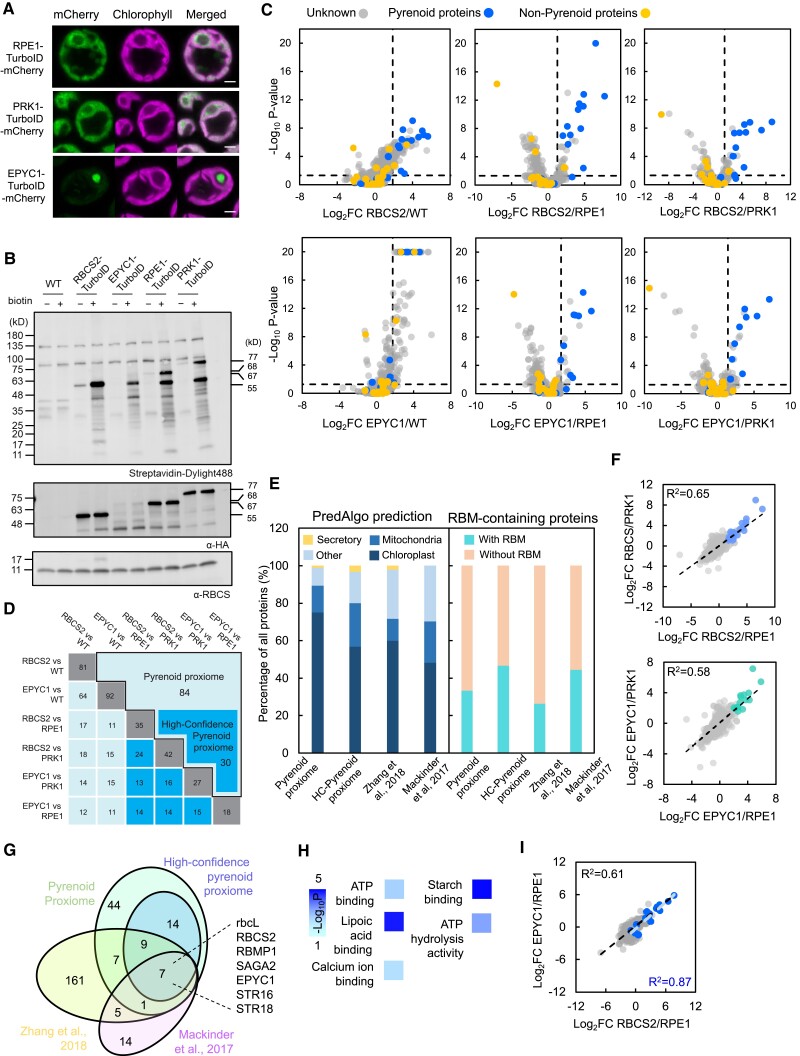
Determining the pyrenoid proteome using proximity labeling. **A)** Localization of the mCherry fusions of RPE1-TurboID, PRK1-TurboID, and EPYC1-TurboID. Green and magenta signals represent mCherry and chlorophyll autofluorescence, respectively. Scale bar is 2 *µ*m. **B)** Labeling activity of RBCS2-TurboID, EPYC1-TurboID, RPE1-TurboID, and PRK1-TurboID strains, as determined in the absence (−) or presence (+) of 2.5 mM biotin for 4 h. Biotinylation was visualized via immunoblotting whole-cell lysate with a streptavidin conjugate. Abundance of RBCS2-TurboID (55 kD), EPYC1-TurboID (68 kD), RPE1-TurboID (67 kD), and PRK1-TurboID (77 kD) was probed by anti-HA. Anti-RBCS was used as a loading control. **C)** Volcano plots representing the Log_2_ FC of RBCS2-TurboID and EPYC1-TurboID compared to WT and stromal controls. Pyrenoid proteins (blue dots) and non-pyrenoid proteins (yellow dots) were used to calculate the enrichment thresholds (vertical dashed line); the values are as follows: RBCS2/WT (1.88); RBCS2/RPE1 (1.31); RBCS2/PRK1 (1.14); EPYC1/WT (1.74); EPYC1/RPE1 (1.67); and EPYC1/PRK1 (1.42). Statistical significance for each pairwise comparison was calculated using the PEAKSQ method, a significance *P*-value cutoff of <0.05 was used (horizontal dashed line). The maximum −Log_10_*P*-value computed by PEAKSQ was 20. **D)** Overlap matrix of identified proteins that are above the enrichment threshold in each treatment group. Bolded border highlights the overall pyrenoid proxiome, while the dark blue shaded box denotes the HC-pyrenoid proxiome. For both the pyrenoid proxiome and HC-pyrenoid proxiome, proteins had to be above the threshold in two or more comparisons. **E)** Predicted localization obtained from PredAlgo (Tardif et al. 2012) and percentage of RBM-containing proteins (Meyer et al. 2020) in the pyrenoid proxiome, the HC-pyrenoid proxiome, and previous published pyrenoid proteomes (Mackinder et al. 2017; Zhan et al. 2018). **F)** Comparison of Log_2_ FC in RBCS2-TurboID and EPYC1-TurboID between the 2 stromal controls. Statistically significant proteins that passed the ROC enrichment threshold are colored. **G)** Venn diagram showing the overlap between the pyrenoid proxiome and HC-pyrenoid proxiome (Mackinder et al. 2017; Zhan et al. 2018). **H)** GO enrichment analysis of the HC-pyrenoid proxiome (*n* = 30) using the PANTHER GO Complete Molecular Function data set. Significance as −Log_10_*P*-value calculated from Fisher's exact test is presented in a color gradient. Only the GO terms of the most specific subclass that were represented by 2 or more proteins are shown. **I)** Comparison of protein enrichment between RBCS2-TurboID and EPYC1-TurboID. Blue dots represent known pyrenoid proteins. The black and blue dashed line represents the calculated trendline using all proteins or known pyrenoid proteins, respectively.

Although our current approach enabled enrichment of pyrenoid proteins, we wished to refine the pyrenoid proteome by trying to distinguish between pyrenoid-specific proteins and proteins that are found within the pyrenoid and the stroma and to remove the bias of increased labeling of abundant background proteins—a typical challenge in proximity labeling studies (Han et al. 2018). To achieve this goal, we developed 2 chloroplast stromal controls and an additional pyrenoid-specific TurboID strain. For stromal controls, we identified 2 Calvin-cycle enzymes, RIBULOSE EPIMERASE 1 (RPE1; encoded by Cre12.g511900) and PHOSPHORIBULOKINASE 1 (PRK1, encoded by Cre12.g554800), which are abundant and localize to the chloroplast stroma but are excluded from the pyrenoid matrix (Fig. 3A) (Küken et al. 2018). We chose the Rubisco linker protein EPYC1 as an additional pyrenoid-specific protein due to its abundance and functional importance for the LLPS of Rubisco to form the pyrenoid (Mackinder et al. 2016). We assembled these new constructs, *RPE1-TurboID*, *PRK1-TurboID*, and *EPYC1-TurboID*, with the *TurboID* cloned in frame at the 3′ end of each coding sequence. We introduced each construct into CC-4533 as above and assessed the activity of the resulting positive strains alongside the RBCS2-TurboID strain (Figs. 3B, S2, A and B, and S3). While we saw evidence for EPYC1-TurboID protein degradation with increased biotin incubation time, the overall biotinylation signal does not appear to be perturbed (Supplemental Fig. S2B). We therefore continued with EPYC1-TurboID with a 4-h biotin incubation time in our MS experiments.

To ensure optimal conditions for identifying the pyrenoid proteome, we grew all expression strains photoautotrophically in 0.04% (*v*/*v*) CO_2_ where nearly all of Rubisco is recruited to the pyrenoid and the CCM is fully induced (Mackinder 2018). Labeling was allowed to proceed for 4 h before we enriched for the resulting biotinylated proteins with streptavidin beads (see Materials and methods). Samples in triplicate were tandem mass tag (TMT) labeled to enable a relative quantification and comparison of protein abundance between each strain (Supplemental Data Set 4). We identified a total of 831 proteins derived from 5,227 peptides, with each protein containing at least 2 unique peptides. We calculated the Log_2_ fold-change (FC) in reporter ion intensity between the pyrenoid-specific TurboID strains (RBCS2-TurboID and EPYC1-TurboID) and controls (WT, RPE1-TurboID, and PRK1-TurboID). We then determined the enrichment of pyrenoid proteins in each comparison. In agreement with our previous pilot experiment, we observed that pyrenoid proteins are predominantly enriched by the pyrenoid-specific TurboID strains across all comparison groups (Fig. 3C, blue dots).

To calculate the enrichment threshold used to assess significant pyrenoid enrichment, we applied the ROC analysis as in Fig. 2, C and D, and a significance threshold of *P* < 0.05 calculated by the PEAKSQ significance test (Cox and Mann 2008). We applied this analysis across all 6 comparison groups (Fig. 3C). This analysis yielded 141 unique proteins across the 6 groups (Supplemental Data Set 5). To remove out possible non-pyrenoid localized proteins, we only considered as true pyrenoid components those identified proteins that were consistently above the enrichment threshold in at least 2 of the comparison groupings. We obtained a final set of 84 unique proteins that we termed the “pyrenoid proxiome” (Fig. 3D, black bordered box). The pyrenoid proxiome contains 14 out of 19 known pyrenoid components detected in our data set and is highly enriched for proteins that are predicted to be targeted to the chloroplast (Fig. 3E).

Next, we set out to see if comparison against stromal control strains improves distinction between pyrenoid proteins and stromal proteins relative to a WT control. We first tested if there were any major differences between our 2 stromal controls. Plotting the Log_2_ FC of RBCS2-TurboID/RPE1-TurboID versus that of RBCS2-TurboID/PRK1-TurboID showed a strong correlation (*R*^2^ = 0.65; Fig. 3F), suggesting that both controls give similar results and that their similar stromal localization is the main driver of protein labeling. We obtained a similar result when comparing EPYC1 against the 2 stromal controls (*R*^2^ = 0.58; Fig. 3F). We next determined the difference between mean Log_2_ FC of known pyrenoid and stromal proteins in each comparison pair (i.e. RBCS2-TurboID vs. WT, RBCS2-TurboID vs. RPE1-TurboID, and so on). Indeed, the difference between mean Log_2_ FC of pyrenoid and stromal proteins was most evident in the stromal control comparisons (Supplemental Fig. S4). This result is further supported by our observation that proteins peripheral to the pyrenoid Rubisco-EPYC1 matrix but not in it, such as LOW-CO_2_-INDUCIBLE PROTEIN B (LCIB), LCIC, STARCH SYNTHASE 2 (STA2), and STARCH BRANCHING ENZYME 3 (SBE3) (Yamano et al. 2010; Mackinder et al. 2017), are not enriched when stromal-specific TurboID strains are used as controls in place of WT. Our data here indicate that using the stromal controls gives a robust proteome of the Rubisco matrix. Taking proteins that are only seen above the threshold in 2 or more comparisons with stromal controls gives us 30 proteins (Supplemental Data Set 6). We named this set the “high-confidence pyrenoid proxiome” (HC-pyrenoid proxiome) (Fig. 3D). Compared to the pyrenoid proxiome, the HC-pyrenoid proxiome contains most known pyrenoid proteins (11/14) found in the former candidate pools. Similar to the abovementioned changes, proteins excluded from the HC-pyrenoid proxiome are either peripheral to the pyrenoid (LCIB and SBE3) or thylakoid membrane proteins (RBMP2). There was also a higher representation of RBM-containing proteins in the HC-pyrenoid proxiome (13/30, ∼43%) than in the pyrenoid proxiome (28/84, ∼28.5%). Our results here give further support for the notion that the use of a compartment control yields a much more precise pyrenoid proxiome. We also evaluated our pyrenoid proxiome against published pyrenoid proteomic data obtained from either pyrenoid purification followed by MS (Zhan et al. 2018) or RBCS1/2 and EPYC1 AP-MS (Mackinder et al. 2017). We determined that 24/84 proteins within the pyrenoid proxiome and 16/30 of the HC-pyrenoid proteome overlap with at least 1 of the published data sets (Fig. 3G). Overall, 7 proteins are present in all 4 data sets, and 5 are known pyrenoid-localized proteins. Taking RBM-containing proteins as a proxy for pyrenoid localization, the HC-pyrenoid proxiome shows the highest fraction of RBM proteins (Fig. 3E). The similarity between our obtained proxiome and previously published data supports proximity labeling as a highly comparable technique to investigate organelle composition.

Gene ontology (GO) term enrichment analysis of the HC-pyrenoid proxiome indicated that these proteins can be functionally grouped into a small number of biological processes (Fig. 3H). These included lipoic acid binding, which represents sulfur-related compounds (GO: GO:0031405), carbohydrate-related processes like alpha-amylase activity and starch-binding (GO: GO:0004556 and GO:2001070, respectively) and ATP-binding groups (GO:0005524). We identified multiple proteins in the HC-pyrenoid proxiome, notably the proteins encoded by Cre06.g269650, Cre03.g158050, and SAGA1 that contain a starch-binding domain alongside a variety of functional domains. This similarity suggests that the matrix–starch interface might act as a specialized site for specific structural or biological functions. A broader analysis of the pyrenoid proxiome also reveals that multiple proteins contain iron–sulfur (Fe-S)-binding domains (encoded by Cre05.g240850, Cre13.g592200, and Cre02.g093650) or have RNA-related functions (encoded by Cre10.g440050, Cre10.g435800, Cre09.g393358, and Cre13.g578650). Tentatively, enrichment of these proteins in the pyrenoid proxiome suggests that the pyrenoid might take on other roles in addition to carbon fixation.

### RBCS-TurboID and EPYC1-TurboID generate comparable pyrenoid proteomes

Rubisco and EPYC1 are the 2 major components of the pyrenoid. Their interactions with each other are both essential for phase separation and pyrenoid formation. However, an AP-MS study using both RBCS2 (and RBCS1) and EPYC1 as baits identified multiple distinct interacting partners as well as a shared set of interactors (Mackinder et al. 2017). Since the majority of pyrenoid proteins we used as benchmark in this study were previously characterized due to their interactions with RBCS, it is difficult to ascertain whether the use of RBCS2-TurboID preferentially labeled Rubisco interactors or the broader pyrenoid proteome. We reasoned that by comparing proteins obtained from RBCS2-TurboID against those with EPYC1-TurboID, we might be able to distinguish between these 2 possibilities and more broadly determine if proximity labeling of proteins in a dynamic molecular condensate preferentially labels the proteome of the condensate or the direct interactors of the bait. A comparison of EPYC1-TurboID and RBCS2-TurboID's respective FC against the stromal controls showed a strong correlation (*R*^2^ = 0.61); this was considerably strengthened when focusing on known pyrenoid proteins (*R*^2^ = 0.87; Fig. 3I, blue dots). We also found 12 out of 15 proteins identified in the EPYC1-TurboID relative to stromal controls (RPE1 and PRK1) in the RBCS2-TurboID relative to stromal controls analysis (Fig. 3D and Supplemental Data Set 5). In conclusion, irrespective of bait, using a mobile protein of the phase-separated pyrenoid yields a high-confidence proteome of the biomolecular condensate.

### Proximity labeling identifies new pyrenoid proteins

**Figure 4.**
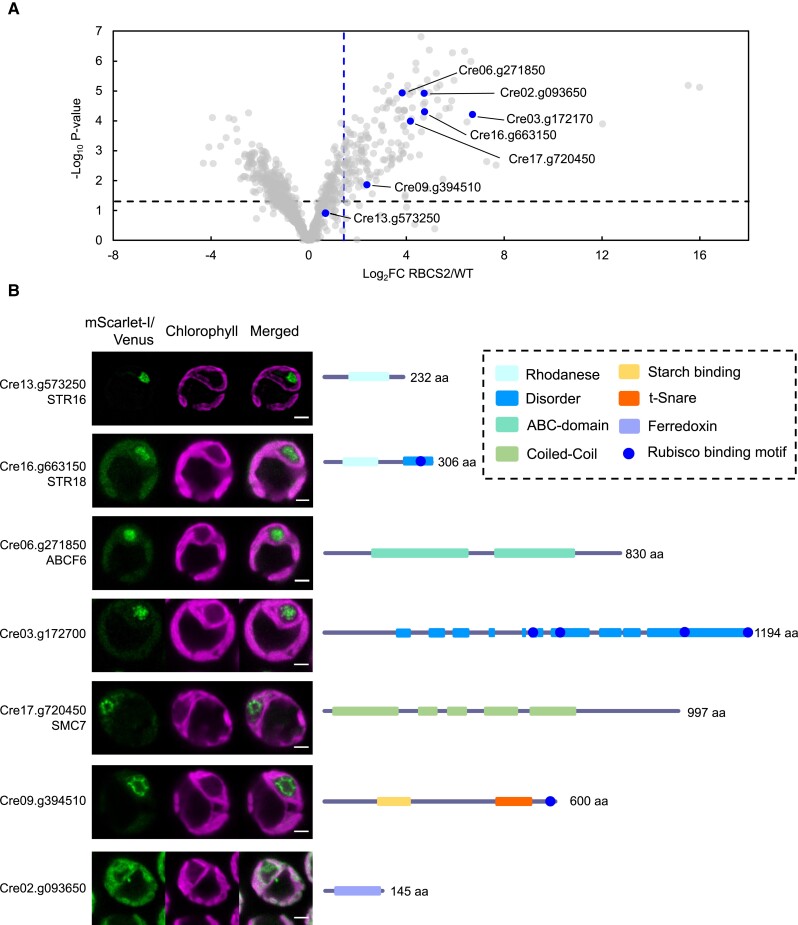
Proximity labeling identifies new pyrenoid proteins. **A)** The volcano plot in Fig. 2B was reproduced here to highlight the proteins that were chosen for localization (blue dots). **B)** Confocal imaging of the chosen proteins. The respective coding regions were cloned in-frame with *Venus* or *mScarlet-I* under their native promoter sequence. Green and magenta signals denote the fluorescence channel and chlorophyll autofluorescence, respectively. Scale bar is 2 *µ*m. Schematic overview of structural prediction from PSI-pred and conserved domains are highlighted next to the confocal images. aa, amino acid.

To validate our pyrenoid proteome, we chose 7 proteins lacking localization data from the preliminary data and our initial RBCS2-TurboID versus WT comparison for fluorescence tagging (Fig. 4A). We primarily selected these proteins based on either occurrence in previous interactome/pull-down data sets (SULFURTRANSFERASE 16 [STR16; Cre13.g573250], STR18 [Cre16.g663150], and ATP-BINDING CASSETTE FAMILY F 6 [ABCF6; Cre06.g271850]; see Fig. 3G) or domain homology to known pyrenoid proteins (Cre03.g172700, Cre09.g394510, and Cre17.g720450). We cloned the open reading frame plus ∼2,000-bp upstream of each target gene in-frame with the sequence encoding the fluorescent proteins Venus or mScarlet-I by recombineering to retain their native promoter (Emrich-Mills et al. 2021). We then transformed each construct into WT *Chlamydomonas*. Six of the 7 tagged proteins showed a primarily pyrenoid localization, with a broad range of subpyrenoid localization patterns (Figs. 4B and S5). Their localization patterns and their domain annotations provide novel insights into pyrenoid function and formation.

STR16, STR18, and ABCF6 showed a localization pattern consistent to the pyrenoid matrix, which is supported by their lack of a predicted transmembrane or starch-binding domain. STR16 and STR18 contain a rhodanese (thiosulfate sulfurtransferase) domain like the previously identified pyrenoid proteins CALCIUM SENSING RECEPTOR 1 (CAS1) and RBMP2. In contrast to STR16 and STR18, CAS1 and RBMP2 lack a critical cysteine in their active site and thus are presumably catalytically inactive. Rhodanese domains have been implicated in an array of functions including disulfide bond formation (Chng et al. 2012) and Fe-S cluster biosynthesis (Bonomi et al. 1977). The latter is particularly interesting as multiple proteins in the pyrenoid proxiome contain an Fe-S cluster domain such as the proteins encoded by Cre02.g093650, Cre08.g365692, and Cre15.g643600 (Supplemental Data Set 5). ABCF6 is predicted to be a member of the ABCF family, which has been shown to regulate protein translation via binding to ribosomes (Boël et al. 2014). The AlphaFold modeling of ABCF6 presents a structure consistent with its ABCF annotation, with the presence of the canonical arm and linker domains (Supplemental Fig. S6; UniProt Consortium 2021; Jumper et al. 2021). A fluorescently tagged version of the protein encoded by Cre03.g172700 formed distinct puncta within the pyrenoid matrix (Figs. 4B and S5) unlike the more homogenous signal observed for matrix proteins such as RBCS2. This subpyrenoid localization suggests that it may be associated with pyrenoid tubules. While PSI-pred structural prediction suggests that the protein encoded by Cre03.g172700 is predominantly disordered, AlphaFold prediction suggests that its C-terminus is composed of a central long alpha-helix surrounded by multiple shorter helices interspaced with disordered sequences that contain 4 RBMs (Figs. 4B and S6). The disordered sequences and RBMs combined might allow the protein encoded by Cre03.g172700 to act as a potential pyrenoid tether that recruits Rubisco to the pyrenoid tubules in a similar fashion as the previously hypothesized function of RBMP1 and RBMP2 (Meyer et al. 2020). Unlike the other proteins that localize to the pyrenoid matrix, STRUCTURAL MAINTENANCE OF CHROMOSOMES 7 (SMC7, encoded by Cre17.g720450) and the protein encoded by Cre09.g394510 are found at the edge of the pyrenoid matrix, with SMC7 forming discrete puncta surrounding the matrix while the protein encoded by Cre09.g394510 appears to line the starch–matrix interface. These proteins show a similar localization pattern as SAGA1, which occupies the starch–matrix–tubule interface. SMC7 lacks the signature ATP-binding and hinge domain important for its predicted function in chromatin condensation (Harvey et al. 2002) and only contains the conserved coiled-coil domain. This structure arrangement mirrors that of SAGA1 and SAGA2 (Itakura et al. 2019) that were also annotated as SMC components and suggests that SMC7 might function in a similar manner. The protein encoded by Cre09.g394510 contains a N-terminal CBM20 starch-binding domain and a t-SNARE domain at its C-terminus, the latter known to mediate vesicle fusion (Han et al. 2017). This observation suggests that the protein encoded by Cre09.g394510 may be involved in membrane remodeling of the pyrenoid tubules, as they are structurally reorganized from thylakoid sheets to pyrenoid tubules as they traverse gaps within the starch sheath (Engel et al. 2015). Collectively, these new pyrenoid proteins represent exciting candidates for further investigation into pyrenoid formation and function.

### Changes in the pyrenoid proteome in response to CO_2_

**Figure 5.**
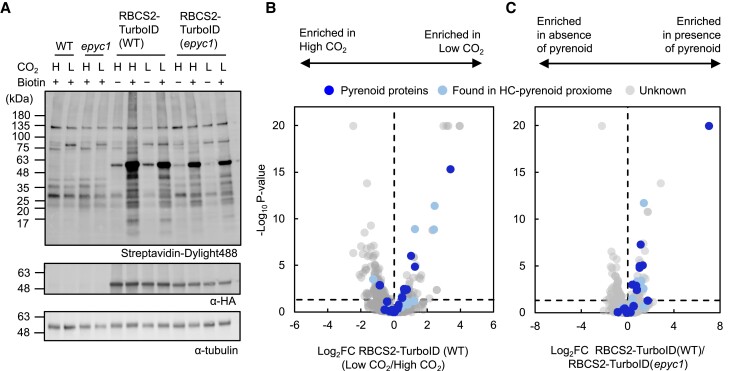
Proximity labeling suggests that the pyrenoid proteome has a subtle response to changes in CO_2_ and phase separation. **A)** Protein labeling of RBCS2-TurboID in WT and *epyc1* as well as their corresponding untagged background were tested under different CO_2_ conditions. Respective strains were grown photoautotrophically and supplemented with 3% CO_2_ (H) or 0.04% CO_2_ (L). Harvested cells were incubated with 2.5 mM biotin for 4 h. Labeling was visualized by immunoblotting the whole cell lysate against streptavidin. Anti-HA was used to probe for RBCS2-TurboID abundance and antitubulin was used as a loading control. **B, C)** Volcano plots representing the Log_2_ FC of RBCS2-TurboID in low CO_2_ versus high CO_2_**B)** or RBCS2-TurboID in the WT background compared to RBCS2-TurboID in the *epyc1* mutant **C)**. Known pyrenoid proteins and the HC-pyrenoid proxiome are colored dark blue and light blue, respectively, while unknowns are colored in gray. Statistical significance for each pairwise comparison was calculated using the PEAKSQ method, a significance cutoff for *P* < 0.05 was used (horizontal dashed line).

When a CCM is not required such as at high CO_2_, the pyrenoid partially dissolves, with ∼50% of Rubisco leaving the pyrenoid into the surrounding stroma (Borkhsenious et al. 1998). In addition, the starch sheath breaks down, and stromal starch content increases (Kuchitsu et al. 1988). However, at a transcriptional and protein abundance level, matrix pyrenoid proteins show a broad range of responses (Brueggeman et al. 2012; Fang et al. 2012; Arias et al. 2020). To explore if the pyrenoid composition changes in response to CO_2_, we compared RBCS2-TurboID strains grown at high and low CO_2_ (Fig. 5, A and B, and Supplemental Data Set 7). Many previously known pyrenoid proteins and proteins in our HC-pyrenoid proxiome were not preferentially enriched across CO_2_ conditions, indicating that the vast majority of the pyrenoid proteome is not CO_2_ responsive. However, a small number of proteins showed a >2 FC, with 20.5% (7/34) enriched at low CO_2_ and 2.9% (1/34) enriched at high CO_2_. Three of the low CO_2_ enriched proteins, SAGA1, LCI9, and AMA3 (ALPHA AMYLASE 3), are associated with starch binding/metabolism. LCI9 was previously localized to the starch plate interfaces and proposed to play a role in starch metabolism (Mackinder et al. 2017). AMA3 is an alpha amylase also involved in starch hydrolysis (Gargouri et al. 2015), and mutants in *SAGA1* have a severe starch structural defect (Itakura et al. 2019). Collectively, these results supports the major remodeling of starch to form the starch sheath under low CO_2_ conditions.

### Possible role of phase separation in protein recruitment to the pyrenoid matrix

The deletion of *EPYC1* leads to abolishment of the pyrenoid and CCM due to the failure to condense Rubisco into the pyrenoid (Mackinder et al. 2016). Confident that the HC-pyrenoid proxiome is labeled by RBCS2-TurboID, we explored how labeling changed when Rubisco was not condensed into the pyrenoid. To this end, we selected RBCS2-TurboID strains in WT and the *epyc1* mutant that accumulate the tagged protein to comparable levels (Fig. 5A). We determined that a large number of known pyrenoid proteins and proteins within the HC-pyrenoid proxiome are enriched in WT when compared to *epyc1* (Fig. 5C and Supplemental Data Set 7), indicating that phase separation either results in more efficient labeling or that phase separation is required for close proximity to Rubisco. However, a subset (11/25) of proteins in the HC-pyrenoid proxiome showed very little enrichment (Log_2_ FC < 0.5) upon Rubisco condensation, suggesting that these proteins may directly interact with Rubisco independently of pyrenoid presence. The differences seen are unlikely due to changes in protein abundance between WT and *epyc1* as these remained highly comparable (Supplemental Fig. S7). Unexpectedly, many proteins containing RBMs required the presence of the pyrenoid to be enriched (top right quadrant of Fig. 5C) indicating that the weak binding affinity (*K*_d_ ∼3 mM; He et al. 2020) of RBMs may not be sufficient to allow Rubisco-RBM complex formation prior to Rubisco condensation by EPYC1.

## Discussion

We established TurboID-based proximity labeling in the chloroplast of the model green alga *C. reinhardtii*. Proximity labeling has proven powerful in unraveling a broad range of cellular functions and suborganelle composition in a diverse range of organisms including plants (Zhang et al. 2019; Mair and Bergmann 2022), diatoms (Turnšek et al. 2021), and cyanobacteria (Dahlgren et al. 2021). However, until now, it had not been established in plastids or *Chlamydomonas*. In parallel to our work, 2 other studies give a snapshot of the diversity of possible applications of TurboID in both plant (Wurzinger et al. 2022) and algal plastids (our study and Kreis et al. 2022). The independently determined similar biotin concentrations and incubation time for labeling in the *Chlamydomonas* chloroplast by our work and the work by Kreis et al. (2022) highlight the reproducibility and robustness of the method.

Once established, we applied TurboID to determine the protein composition of the phase-separated pyrenoid. We identified a “pyrenoid proxiome” containing 84 proteins. A large number of previously localized pyrenoid proteins (67%) from the literature were present in our pyrenoid proxiome. However, it did miss several previously classified pyrenoid proteins. A deeper analysis of these missing proteins indicated that they were primarily located within specific pyrenoid subcompartments where they may remain inaccessible by the matrix generated biotin radicals. For example, CARBONIC ANHYDRASE 3 (CAH3), a pyrenoid tubule lumen protein, was not among the identified proteins in our proxiome most likely due to the limited penetration of biotin radicals across membranes (Rhee et al. 2013).

By including robust stromal controls for proteins that are adjacent to the pyrenoid but do not partition into the matrix, we established a “HC-pyrenoid proxiome” containing 30 proteins. This protein set excluded multiple proteins classified as pyrenoid proteins that are found at the pyrenoid periphery but do not partition into the matrix. These proteins included LCIB, LCI9, LCIC, and SBE3. These data along with the identification of nearly all known matrix proteins and proteins with RBMs that are at the matrix interface (i.e. SAGA1, BST4, and RBMP2) give us high confidence in this data set.

GO term enrichment analysis of the HC-pyrenoid proxiome and a broader analysis of the pyrenoid proxiome showed the enrichment of proteins in a small number of biochemical functions and pathways, suggesting that the pyrenoid plays additional roles to CO_2_ concentration. Three groups that stood out were RNA-binding/translation proteins, Fe-S-containing proteins, and starch-binding proteins. Biomolecular condensates are regularly associated with RNA sequestration and processing (Banani et al. 2017). This association allows cells to respond in a timely manner in face of cellular stress. In *Chlamydomonas*, the photosynthetic machinery is translated at a specialized position adjacent to the pyrenoid called the translation zone (or T-zone; Sun et al. 2019). Under light and oxidative stress, the mRNA of the core photosystem II component PsbA becomes enriched within the pyrenoid matrix (Uniacke and Zerges 2008; Zhan et al. 2015), which suggests that the pyrenoid recruits RNA as a stress response. However, the molecular basis and function of this mRNA sequestration remains unclear. In this study, we identified multiple RNA-associated proteins within the pyrenoid proxiome (proteins encoded by Cre10.g440050, Cre10.g435800, Cre09.g393358, and Cre13.g578650). We also localized a new ribosome-associated protein, ABCF6, to the pyrenoid. An *Escherichia coli* homolog of ABCF6, EttA, was demonstrated to prevent translation by its binding to 70S ribosomes in a ATP/ADP ratio-dependent manner (Boël et al. 2014). The localization of ABCF6 to the pyrenoid further supports a role for the pyrenoid in RNA metabolism, by either sequestering chloroplast ribosomes in the pyrenoid or partitioning ABCF6 away from chloroplast ribosomes under certain environmental conditions.

Fe-S protein assembly and activity is typically sensitive to molecular O_2_ (Boyd et al. 2014). It was intriguing to see that the pyrenoid was enriched for both Fe-S assembly and Fe-S-containing proteins. A proposed, but unconfirmed, function of the pyrenoid to enhance CO_2_ fixation is to minimize the presence of O_2_ to increase the CO_2_:O_2_ ratio at the active site of Rubisco. A reduced O_2_ environment could also favor other O_2_-sensitive biological reactions. We found that the rhodanese domain-containing proteins STR16 and STR18 are localized to the pyrenoid; rhodanese domains are linked to the biogenesis of Fe-S clusters (Rydz et al. 2021). Pyrenoid localization might allow them to be shielded from the oxygenic environment outside the pyrenoid matrix, allowing these oxygen-sensitive reactions to be carried out. Alternatively, rhodanese has also been suggested to participate in reactive oxygen species (ROS) scavenging via the production of reactive sulfur species (Wang et al. 2021). Since ROS have also been found to drive pyrenoid formation (Neofotis et al. 2021), the presence of rhodanese domain-containing proteins in the pyrenoid suggests that the pyrenoid itself is involved with ROS metabolism or redox signaling.

The pyrenoid starch sheath is proposed to act as a diffusion barrier that limits CO_2_ diffusion away from the pyrenoid matrix. Recent evidence has suggested that this matrix–starch association is critical for the organization of many pyrenoid components. The deletion of the gene encoding the starch-binding protein SAGA1 results in the formation of multiple pyrenoids with altered starch sheath and pyrenoid tubule morphology (Itakura et al. 2019). Additionally, the knockout of *ISOAMYLASE 1* (*ISA1*) that abolishes the pyrenoid starch sheath results in the CCM-essential carbonic anhydrase LCIB to mislocalize as an aggregate at the basal region of the pyrenoid (Toyokawa et al. 2020), in contrast to its typical pyrenoid peripheral localization. Together, starch-binding proteins are crucial to the functioning of the pyrenoid in CCM-related functions. In this work, we localized an additional protein (encoded by Cre09.g394510 and contained a starch-binding CBM20 domain) to the pyrenoid. This protein contains an additional t-SNARE functional domain and has a similar domain arrangement to SAGA1 and LCI9, which also share a similar localization pattern (Mackinder et al. 2017). Investigating the role of these proteins in pyrenoid structural organization and function may provide insights into pyrenoid assembly needed for future engineering of a functional pyrenoid into land plants (Adler et al. 2022).

Once we had determined a HC-pyrenoid proteome, we explored the change in the proxiome of Rubisco at low or high CO_2_ and with (WT) or without (*epyc1*) phase separation. Surprisingly, most proteins appeared to be present in the pyrenoid under both CO_2_ conditions, indicating that the core proteome of the pyrenoid is relatively stable. However, a subset involved in starch metabolism was predominantly enriched under low CO_2_ when starch needs to be remodeled to form a CO_2_ leakage barrier. By using the *epyc1* mutant, we explored how labeling by RBCS2-TurboID differs when Rubisco condensation into the pyrenoid is disrupted. Most HC-pyrenoid proxiome components were enriched by Rubisco condensation, indicating that they are brought into closer proximity upon pyrenoid formation. However, a subset showed very little change, suggesting that they may already be interacting with Rubisco independently of pyrenoid assembly. For both the high versus low CO_2_ and WT versus *epyc1* comparisons, it should be noted that the 4 h incubation time of labeled strains could have led to translational changes resulting in compounding data between absolute protein amounts and partitioning into the pyrenoid. In addition, the partial dissolution of the pyrenoid during high CO_2_ also resulted in a higher proportion of RBCS2-TurboID in the dilute phase. This in turn potentially increases labeling of proteins that have not yet partitioned into the pyrenoid. In the future, shorter labeling times may help further refine the pyrenoid proteome under varying conditions.

Proximity labeling has been underutilized for understanding phase-separated proteomes that are highly dynamic and thus are challenging to purify (Hubstenberger et al. 2017). The presence and exchange of bait proteins between the condensed phase and dilute phase might result in reduced specificity of RBCS2/EPYC1-TurboID over time and labeling outside of the condensate. To counteract this issue, we found that the use of abundant soluble controls that are excluded from the pyrenoid allowed the determination of a highly refined pyrenoid proteome. Future experiments using proximity labeling, specifically to determine the proteomes of biomolecular condensates, should include carefully chosen controls.

To make TurboID easily accessible for other laboratories using *Chlamydomonas*, we based our constructs on the MoClo golden gate cloning framework that enables TurboID to be used with a broad range of parts (Crozet et al. 2018) and easily fused to proteins that are already within this framework. To enable easy adoption of this powerful method, all developed vectors and lines were deposited at the Chlamydomonas Resource Center.

## Materials and methods

### Construction of APEX2/TurboID vectors in *C. reinhardtii*

Construction of *APEX2/TurboID*-expression cassettes for *Chlamydomonas* was designed using the MoClo system Chlamydomonas MoClo toolkit (Crozet et al. 2018). Golden Gate-compatible syntaxes were added to synthesize parts encoding the APEX2/TurboID enzyme and target proteins (RBCS2/EPYC1) or via PCR using CC-4533 genomic DNA for RPE1/PRK1 (see Supplemental Data Set 9 for all primer sequences used). Due to the low complexity and high repeat nature of EPYC1, the *EPYC1* coding sequence was synthesized in 4 parts as a Level-1 construct, while the *RBCS2* coding sequence was synthesized as 2 parts to avoid a detected sequence repeat. The *APEX2* and *TurboID* tag sequences (Branon et al. 2018; Ganapathy et al. 2018) were codon optimized for *Chlamydomonas* (Nakamura et al. 2000) with the *RBCS2i2* (Cre02.g120150) and *LHCBM1i2* (Cre01.g066917) introns inserted at ∼500-bp increments to improve protein production (Baier et al. 2018). The coding sequence of the tags was similarly synthesized as Level-1 parts. Together, the Level-1 and PCR-amplified target genes, *APEX2/TurboID* tag, and a sequence encoding a small flexible linker (GSGSTSGSGS) were assembled to a Level-0 product occupying the B3-B4 MoClo position using the pUAP1 backbone such that the target genes are expressed with the sequence encoding the enzyme tag at their 3′ end, bridged by the small flexible linker. The Level-1 cassette was then assembled using the target gene-*TurboID/APEX2* fusion part, the *PSAD* promoter/terminator pair, and either a tandem HA/Flag tag epitope at the 3′ end of the construct for labeling experiments or a sequence encoding mCherry for localization. The resultant Level-2 expression module consists of the target gene-*TurboID* fusion cassette and an antibiotic resistance cassette for selection. To enable accessible use of TurboID-based proximity labeling in the Golden Gate cloning pipeline, the identical *TurboID* coding sequence with the flexible linker was also cloned into a Level-0 part occupying the B4 MoClo position. Sequences for all developed vectors are in Supplemental Data Set 1. All vectors and strains are deposited at the Chlamydomonas Resource Center (https://www.chlamycollection.org).

### *Chlamydomonas* growth and transformation

*Chlamydomonas* cultures were maintained on TAP medium with revised Hunter's trace elements (Kropat et al. 2011). For biotin labeling experiments, cells were grown photoautotrophically in Tris phosphate (TP) medium at ∼21 °C under LED lights (Valoya C65 LEDs with AP673L spectrum) at ∼50 *µ*mol photons m^−2^ s^−1^. Assembled plasmids were linearized with I-SceI (for fluorescent tagging plasmids) or BsaI (for proximity labeling plasmids) and transformed into *Chlamydomonas* via electroporation according to (Mackinder et al. 2017).

### Protein extraction and immunoblotting

For immunoblotting, cells were grown photoautotrophically to mid-log phase and were harvested by centrifugation 17,900 × *g* for 5 min at 4 °C. Cell pellets were resuspended in lysis buffer (25 mM Tris–HCl pH 7.4, 300 mM NaCl, 1 mM DTT, 5 mM MgCl_2_, 0.1 mM PMSF, 1× EDTA-free protease inhibitor [Roche], 0.1% [*w*/*v*] SDS, 0.5% [*w*/*v*] deoxycholic acid, and 1% [*v*/*v*] Triton X-100) before snap-freezing in liquid nitrogen. The cell suspensions were lysed by 5 freeze/thaw cycles and centrifuged at 17,900 × *g* for 10 min at 4 °C. The resulting supernatants were used as protein samples in later experiments and stored at −70 °C if not used immediately. For immunoblotting, boiled protein samples were resolved by SDS–PAGE and transferred to a PVDF membrane via a semidry transfer system. Membrane was blocked with 3% (*w*/*v*) BSA in Tris-buffered saline with 0.1% (*v*/*v*) Tween 20 (TBST) and probed with antibodies accordingly. Antibodies were diluted in TBST as follows: Streptavidin Dylight-488 conjugate (1:4,000, Fisher Scientific #21832); anti-HA (1:1,000, Fisher Scientific 26183); anti-Flag (1:1,000, Sigma #F1804); and antitubulin (1:2,000, Sigma #T6074).

### Biotin labeling and streptavidin affinity purification

All 3 TurboID-labeling experiments were performed similarly. The starter culture of TurboID expression strains and WT were grown to mid-log phase in TAP medium. They were used to inoculate 400 mL of TP medium supplied with elevated CO_2_ (3% [*v*/*v*] CO_2_) until mid-log phase and then transferred to air-level CO_2_ (0.04% [*v*/*v*] CO_2_) for ∼2 d or maintained at 3% (*v*/*v*) CO_2_ as indicated. Cells were harvested by centrifugation 1,500 × *g* for 5 min at room temperature. They were then resuspended in fresh TP medium in a 6-well cell culture plate to an OD_750_ of 2.5. Then, 100 mM biotin stock in DMSO was added to the cell suspension to a final concentration of 2.5 mM to initiate the labeling reaction. Biotin labeling was allowed to proceed for 1 to 8 h in the pilot experiment or for 4 h in the later experiments on an orbital shaker. Biotin-labeled cells were harvested by centrifugation 21,300 × *g*, 2 min at 4 °C and rinsed 3 times with ice-cold TP medium. Cell pellets were snap frozen in liquid nitrogen and stored at −70 °C until streptavidin affinity purification.

For APEX2 labeling, the RBCS2-APEX2 expression cells were grown and harvested to an OD_750_ of 2.5 as mentioned above. Biotin-phenol at a final concentration of 2.5 mM was added to the harvested cell suspension from a 250 mM biotin-phenol stock in DMSO. Biotin-phenol incubation was performed for 2 h on an orbital shaker at 20, 30, or 37 °C. The H_2_O_2_ activator at 2 mM concentration was spiked into the suspension to initiate biotin labeling for 2 min. The reaction was then quenched by addition of an ice-cold quencher solution (10 mM sodium ascorbate, 5 mM Trolox, and 10 mM sodium azide in PBS, pH 7.4) and pelleted by centrifugation 21,300 × *g* for 1 min at 4 °C and stored at −70 °C until streptavidin affinity purification.

Protein extraction was carried out as described above. Prior to streptavidin affinity pull-down, free biotin was removed from protein samples using a Zeba Spin Desalting column (#89891, Thermo Fisher) using lysis buffer. To determine protein concentration, a small aliquot (50 *µ*L) of the desalted protein was diluted 10 times in water, and concentration was measured using a Pierce BCA protein assay kit (#23225, Thermo Fisher) as per the manufacturer's instructions. For streptavidin affinity purification, a total of 1.75 mg of protein was used with 50 *µ*L of Pierce Streptavidin Magnetic Beads (88816; Thermo Fisher) equilibrated with lysis buffer. The bead suspension was incubated at 4 °C overnight on a rotor wheel. Beads were then washed twice with lysis buffer for 5 min: once with 1 M KCl for 2 min; once with 0.1 M NaCO_3_ for 1 min; once with 4 M urea in 50 mM triethylammonium bicarbonate, pH 8.5 (TEAB) for 1 min; once with 6 M urea in 50 mM TEAB for 1 min; and twice with 50 mM TEAB buffer for 5 min. Washed beads were frozen at −70 °C until submitted for mass spectrometry.

### LC-MS/MS and analysis of APEX2 and TurboID pilot studies

#### APEX2 digestion

For the APEX2 experiments, streptavidin beads were eluted by boiling with 2× Laemmli loading buffer (Biorad, 161 to 0737) containing 20 mM DTT and 2 mM biotin. The eluate was then run on a 4% to 15% Tris-glycine gel (Biorad, #4561084) for 30 min at 50 V. Gel slices were then fixed according to Mackinder et al. (2017). In-gel tryptic digestion was performed after reduction with 10 mM dithioerythritol and 50 mM *S*-carbamidomethylation with iodoacetamide. Gel pieces were washed 2 times with aqueous 50% (*v*/*v*) acetonitrile containing 25 mM ammonium bicarbonate and then once with acetonitrile and dried in a vacuum concentrator for 20 min. A 500-ng aliquot of sequencing-grade trypsin (Promega) was added prior to incubation at 37 °C for 16 h.

#### TurboID digestion

For the TurboID pilot experiment, on-bead digestion was performed after reduction with 10 mM tris(2-carboxyethyl)phosphine and alkylation with 10 mM iodoacetamide in 50 mM TEAB containing 0.01% (*w*/*v*) ProteaseMAX surfactant (Promega). A 500-ng aliquot of sequencing-grade trypsin (Promega) was added prior to incubation at 37 °C for 16 h.

#### LC-MS/MS acquisition of APEX2 and TurboID pilot experiments

Resulting peptides were resuspended in aqueous 0.1% (*v*/*v*) trifluoroacetic acid and then loaded onto an mClass nanoflow UPLC system (Waters) equipped with a nanoEaze M/Z Symmetry 100-Å C_18_ and 5-*µ*m trap column (180 *µ*m × 20 mm, Waters) and a PepMap, 2-*µ*m, 100-Å, and C_18_ EasyNano nanocapillary column (75 mm × 500 mm, Thermo). The trap wash solvent was aqueous 0.05% (*v*/*v*) trifluoroacetic acid and the trapping flow rate was 15 *µ*L/min. The trap was washed for 5 min before switching the flow to the capillary column. Separation used gradient elution of 2 solvents: solvent A, aqueous 0.1% (*v*/*v*) formic acid; and solvent B, acetonitrile containing 0.1% (*v*/*v*) formic acid. The flow rate for the capillary column was 300 nL/min, and the column temperature was 40 °C. The linear multistep gradient profile was 3% to 10% B over 7 min, 10% to 35% B over 80 min, and 35% to 99% B over 10 min and then proceeded to wash with 99% solvent B for 8 min. The column was returned to initial conditions and reequilibrated for 15 min before subsequent injections. The nanoLC system was interfaced with an Orbitrap Fusion Tribrid mass spectrometer (Thermo) with an EasyNano ionization source (Thermo). Positive ESI-MS and MS^2^ spectra were acquired using Xcalibur software (version 4.0, Thermo). Instrument source settings were ion spray voltage, 1,900 V; sweep gas, 0 Arb; ion transfer tube temperature; and 275 °C. MS^1^ spectra were acquired in the Orbitrap with the following: 120,000 resolution, scan range: *m/z* 375 to 1,500; AGC target, 4e^5^; and max fill time, 100 ms. Data-dependent acquisition was performed in top speed mode using a 1-s cycle, selecting the most intense precursors with charge states >1. Easy-IC was used for internal calibration. Dynamic exclusion was performed for 50-s postprecursor selection, and a minimum threshold for fragmentation was set to 5 × 10^3^. MS^2^ spectra were acquired in the linear ion trap with the following: scan rate, turbo; quadrupole isolation, 1.6 *m/z*; activation type, HCD; activation energy, 32%; AGC target, 5 × 10^3^; first mass, 110 *m/z*; and max fill time, 100 ms. Acquisitions were arranged by Xcalibur to inject ions for all available parallelizable time.

#### Spectral counting APEX2

Peak lists in Thermo.raw format were converted to .mgf using MSConvert (version 3.0, ProteoWizard) before submitting to database searching against 19,716 *Chlamydomonas* protein sequences appended with common proteomic contaminants. Mascot Daemon (version 2.6.0, Matrix Science) was used to submit the search to a locally running copy of the Mascot program (Matrix Science Ltd., version 2.7.0). Mascot was searched with a fragment ion mass tolerance of 0.50 D and a parent ion tolerance of 3.0 ppm. O-124 of pyrrolysine, j-16 of leucine/isoleucine indecision, and carbamidomethyl of cysteine were specified in Mascot as fixed modifications. Oxidation of methionine was specified in Mascot as a variable modification. Scaffold (version Scaffold_5.2.0, Proteome Software Inc., Portland, OR, USA) was used to validate MS/MS-based peptide and protein identifications. Peptide identifications were accepted if they could be established at >84.0% probability to achieve a false discovery rate (FDR) of 1.0% or less by the percolator posterior error probability calculation. Protein identifications were accepted if they could be established at >6.0% probability to achieve an FDR of <1.0% and contained at least 2 identified peptides. Quantitative value of total spectra was used to calculate the Log_2_ FC between RBCS2-APEX2 and WT samples, and the Student's *t*-test derived *P*-value was −Log_10_ transformed before presented.

#### Precursor intensity-based relative quantification TurboID pilot

Peak lists in .raw format were imported into Progenesis QI (version 2.2., Waters) and LC-MS runs aligned to the common sample pool. Precursor ion intensities were normalized against total intensity for each acquisition. A combined peak list was exported in .mgf format for database searching against 19,716 *Chlamydomonas* protein sequences appended with common proteomic contaminants. Mascot Daemon (version 2.6.0, Matrix Science) was used to submit the search to a locally running copy of the Mascot program (Matrix Science Ltd., version 2.7.0). Search criteria specified were as follows: enzyme, trypsin; max missed cleavages, 1; fixed modifications, carbamidomethyl (C); variable modifications, oxidation (M); peptide tolerance, 3 ppm; MS/MS tolerance, 0.5 D; and instrument, ESI-TRAP. Peptide identifications were passed through the percolator algorithm to achieve a 1% FDR assessed against a reverse database and individual matches filtered to require minimum expect score of 0.05. The Mascot .XML result file was imported into Progenesis QI and peptide identifications associated with precursor peak areas matched between runs. Relative protein abundance was calculated using precursor ion areas from nonconflicting unique peptides. Accepted protein quantifications were set to require a minimum of 2 unique peptide sequences. Missing values were then replaced by the minimal value detected from each bait. The FC in the RBCS2-TurboID versus WT comparison was calculated on the sum of relative protein abundance at all time points and was Log_2_ transformed. Statistical testing was performed in Progenesis QI from ArcSinh-normalized peptide abundances and the ANOVA-derived *P*-values was −Log_10_ transformed and presented.

### LC-MS/MS and analysis of TMT-labeled TurboID experiments

#### TurboID digestion and TMT labeling

For the TurboID experiments in Figs. 3 and 4, on-bead digestion was performed after reduction with 10 mM tris(2-carboxyethyl)phosphine and alkylation with 50 mM methyl methanethiosulfonatein in 50 mM TEAB. A 500-ng aliquot of sequencing-grade trypsin (Promega) was added prior to incubation at 37 °C for 16 h. Postdigestion, the peptide-containing supernatants were removed from the beads for TMT labeling. Peptides were labeled with TMTPro 16-plex reagents (Thermo Fisher) as detailed in the manufacturer's protocol. Postlabeling samples were combined and dried in a vacuum concentrator before reconstituting in 100-mL H_2_O.

#### LC-MS/MS acquisition of TMT-labeled TurboID experiment

Peptides were fractionated by high pH reversed phase C_18_ HPLC. Samples were loaded onto an Agilent 1260 II HPLC system equipped with a Waters XBridge 3.5-*µ*m, C_18_ column (2.1 mm × 150 mm, Thermo). Separation used gradient elution of 2 solvents: solvent A, aqueous 0.1% (*v*/*v*) ammonium hydroxide; and solvent B, acetonitrile containing 0.1% (*v*/*v*) ammonium hydroxide. The flow rate for the capillary column was 200 mL/min, and the column temperature was 40 °C. The linear multistep gradient profile for the elution was 5% to 35% B over 20 min and 35% to 80% B over 5 min; the gradient was followed by washing with 80% (*v*/*v*) solvent B for 5 min before returning to initial conditions and reequilibrating for 7 min prior to subsequent injections. Eluate was collected at 1-min intervals into LoBind Eppendorf tubes. Peptide elution was monitored by UV absorbance at 215 and 280 nm. Fractions were pooled across the UV elution profile to give 12 fractions for LC-MS/MS acquisition. Peptide fractions were dried in a vacuum concentrator before reconstituting in 20 mL aqueous 0.1% (*v*/*v*) trifluoroacetic acid.

TMT-labeled peptides fractions were loaded onto an mClass nanoflow UPLC system (Waters) equipped with a nanoEaze M/Z Symmetry 100-Å C_18_ and 5-*µ*m trap column (180 *µ*m × 20 mm, Waters) and a PepMap, 2-*µ*m, 100-Å, and C_18_ EasyNano nanocapillary column (75 mm × 500 mm, Thermo). The trap wash solvent was aqueous 0.05% (*v*/*v*) trifluoroacetic acid, and the trapping flow rate was 15 *µ*L/min. The trap was washed for 5 min before switching the flow to the capillary column. Separation used gradient elution of 2 solvents: solvent A, aqueous 0.1% (*v*/*v*) formic acid; and solvent B, acetonitrile containing 0.1% (*v*/*v*) formic acid. The flow rate for the capillary column was 330 nL/min, and the column temperature was 40 °C. The linear multistep gradient profile was 2.5% to 10% B over 10 min, 10% to 35% B over 75 min, and 35% to 99% B over 15 min before proceeded to wash with 99% solvent B for 5 min. The column was returned to initial conditions and reequilibrated for 15 min before subsequent injections. The nanoLC system was interfaced to an Orbitrap Fusion hybrid mass spectrometer (Thermo) with an EasyNano ionization source (Thermo). Positive ESI-MS, MS^2^, and MS^3^ spectra were acquired using Xcalibur software (version 4.0, Thermo). Instrument source settings were as follows: ion spray voltage, 2,100 V; sweep gas, 0 Arb; and ion transfer tube temperature, 275 °C. MS^1^ spectra were acquired in the Orbitrap with: 120,000 resolution, scan range; *m/z* 380 to 1,500; AGC target, 2 × 10^5^; and max fill time, 50 ms. Data-dependent acquisition was performed in top speed mode using a 4-s cycle, selecting the most intense precursors with charge states 2 to 6. Dynamic exclusion was performed for 50-s postprecursor selection, and a minimum threshold for fragmentation was set at 3 × 10^4^. MS^2^ spectra were acquired in the linear ion trap with: scan rate, turbo; quadrupole isolation, 1.2 *m/z*; activation type, CID; activation energy, 35%; AGC target, 1 × 10^4^; first mass, 120 *m/z*; and max fill time, 35 ms. MS^3^ spectra were acquired in multinotch synchronous precursor mode (SPS^3^), selecting the 5 most intense MS^2^ fragment ions between 400 and 1,000 *m/z*. SPS^3^ spectra were measured in the Orbitrap mass analyzer using 50,000 resolution; quadrupole isolation, 1 *m/z*; activation type, HCD; collision energy, 65%; scan range, *m/z* 110 to 500; AGC target, 4 × 10^5^; and max fill time, 10 ms. Acquisitions were arranged by Xcalibur to inject ions for all available parallelizable time.

#### Protein identification and TMT label intensity quantification

Peak lists in .raw format were imported into PEAKS StudioX Pro (version 10.6 Bioinformatics Solutions Inc.) for peak picking, database searching, and relative quantification. MS2 peak lists were searched against 19,716 *Chlamydomonas* protein sequences appended with common proteomic contaminants. Search criteria specified were as follows: enzyme, trypsin; max missed cleavages, 1; fixed modifications, TMT16plex (K- and N-term peptide); variable modifications, oxidation (M); peptide tolerance, 3 ppm; MS/MS tolerance, 0.5 D; and instrument, ESI-TRAP. Peptide identifications were filtered to achieve a 1% peptide spectral match FDR as assessed empirically against a reversed database search. Protein identifications were further filtered to require a minimum of 2 unique peptides per protein. TMT reporter ion intensities acting as markers of relative intersample peptide abundance were extracted from MS^3^ spectra for quantitative comparison. Protein level quantification significance used ANOVA for multiway comparison and the PEAKSQ significance test for pairwise comparisons. In both cases, the null hypothesis was that individual protein abundance was equal between groups. Normalization of label intensity was then carried out using the global ratio derived from total intensity of all labels. The FCs between comparison groups were calculated based on their normalized TMT reporter ion intensities. Proteins that were not detected in all replicates for an individual bait were removed from calculation. Missing values were then replaced by the minimal value detected from each bait. Significance was determined via PEAKSQ test represented as −Log_10_*P* value.

### Recombineering cloning for localization

Cloning of fluorescent protein-tagged constructs was performed as previously described (Emrich-Mills et al. 2021). Briefly, homology arms to target genes at the 5′ of the native promoter and 3′ UTR were added to destination vectors via PCR. Homology arms of Cre13.g573250 were cloned into the pLM162-mScarlet-I backbone. Homology arms of Cre16.g663150, Cre06.g271850, Cre03.g172700, Cre17.g720450, Cre09.g394510, and Cre02.g093650 were cloned into the pLM099-Venus backbone. Amplified backbones were transformed by electroporation into *E. coli* containing a bacterial artificial chromosome and RecA vector, which drives the recombination event. The resulting plasmids were selected on LB agar plates containing kanamycin and junctions confirmed by sequencing.

### Imaging of fluorescently tagged lines

For imaging of fluorescently tagged lines, photoautotrophically grown cells were immobilized on 1.5% (*w*/*v*) low-melting point agarose in TP medium. Indirect immunofluorescence of RBCS2-APEX2 was performed according to Uniacke et al. (2011) with the following modifications: cells were fixed with 3.7% (*w*/*v*) formaldehyde solution in PBS for 30 min at room temperature. Anti-Flag antibody (F1804; Sigma-Aldrich) at 1:1,000 dilution in PBS containing 1% (*w*/*v*) BSA was used as primary antibody. Anti-Mouse Alexa Fluor plus 555 (A32727; Invitrogen) was used as the secondary antibody at 1:1,000 dilution. Labeled cells were then kept in the dark prior to imaging. Images were taken using a Zeiss LSM880 microscope with the Airyscan module or a Zeiss Elyra7 Lattice SIM. Excitation and emission filters of fluorophore and chlorophyll autofluorescence were set as follows: mVenus (excitation: 514 nm; emission: 520 to 550 nm); chlorophyll (excitation: 633; emission: 610 to 650 nm); and mCherry/mScarlet-I/Alexa Fluor plus 555 (excitation: 561 nm; emission: 580 to 600 nm).

### Amplex Red assay

Amplex UltraRed assay for RBCS2-APEX2 peroxidase activity was carried out according to the manufacturer’s manual. Briefly, Amplex Red reagent (Fisher Scientific; Invitrogen Amplex UltraRed Reagent #10737474) was dissolved in DMSO to a 10 mM stock. RBCS2-APEX2 and the untagged WT strains were grown photoautotrophically and split into triplicates. Cells were then chilled on ice for 5 min before resuspending in 200 *µ*L of reaction buffer (50 M Amplex Red, 2 mM H_2_O_2_ in PBS, and pH 7.4). The reaction was carried out on ice for 15 min. Resorufin fluorescence measurement was performed using a Clariostar Plus Microplate reader using the following excitation and emission settings: resorufin (excitation: 535 to 555 nm; emission: 580 to 620 nm) and chlorophyll autofluorescence (excitation: 610 to 630 nm; emission: 660 to 695 nm).

### Accession numbers

Sequence data from this article can be found in Phytozome, the Plant Comparative Genomics portal of the Department of Energy's Joint Genome Institute, under the following accession numbers: Cre02.g120150: RBCS2; Cre10.g436550: EPYC1; Cre12.g511900: RPE1; Cre12.g554800: PRK1; Cre13.g573250: STR16; Cre16.g663150: STR18; Cre06.g271850: ABCF6; Cre03.g172700; Cre17.g720450: SMC7; Cre09.g394510; and Cre02.g093650. Proteomic data are deposited in MassIVE: https://doi.org/doi:10.25345/C5057D306 with ProteomeXchange identifier: PXD041970.

## Supplementary Material

koad131_Supplementary_Data

## Supplemental data

The following materials are available in the online version of this article.

**Supplemental Figure S1**. APEX2 does not efficiently label pyrenoid proteins in the *Chlamydomonas* chloroplast.

**Supplemental Figure S2**. Screening strains for TurboID accumulation and activity.

**Supplemental Figure S3**. Confocal imaging of mCherry tagged TurboID strains.

**Supplemental Figure S4**. Enrichment of differentially localized proteins using different controls.

**Supplemental Figure S5**. Additional images of the TurboID identified pyrenoid proteins.

**Supplemental Figure S6**. AlphaFold-modeled structures for the proteins encoded by Cre06.g271850 (ABCF6) and Cre03.g172700.

**Supplemental Figure S7**. Comparison of protein abundance between WT and *epyc1* background strains expressing *RBCS2-TurboID* after streptavidin affinity purification.

**Supplemental Data Set 1**. A list of all plasmids used and developed in this study.

**Supplemental Data Set 2**. Pilot mass spectrometry analysis of RBCS2-TurboID samples against WT controls.

**Supplemental Data Set 3**. Localization of proteins based on fluorescence tagging/immunofluorescence obtained from the literature.

**Supplemental Data Set 4**. TMT mass spectrometry analysis of pyrenoid baits against stromal and WT controls.

**Supplemental Data Set 5**. Pyrenoid proxiome.

**Supplemental Data Set 6**. HC-pyrenoid proxiome.

**Supplemental Data Set 7**. TMT mass spectrometry analysis of RBCS2-TurboID at different CO_2_ levels and in the *epyc1* mutant.

**Supplemental Data Set 8**. The statistical summary of results shown in Fig. 2E.

**Supplemental Data Set 9**. Oligo sequences used.
