# Supplementary material for: A phase-separated CO2-fixing pyrenoid proteome determined by TurboID in Chlamydomonas reinhardtii
Source: Plant Cell. 2023 May 17;35(9):3260–79. doi: 10.1093/plcell/koad131 (PMC10473203; doi:10.1093/plcell/koad131)
Supplement: koad131_Supplementary_Data [file koad131_supplementary_data.zip › tpc.22.01174_Supplemental_Figures.pdf]

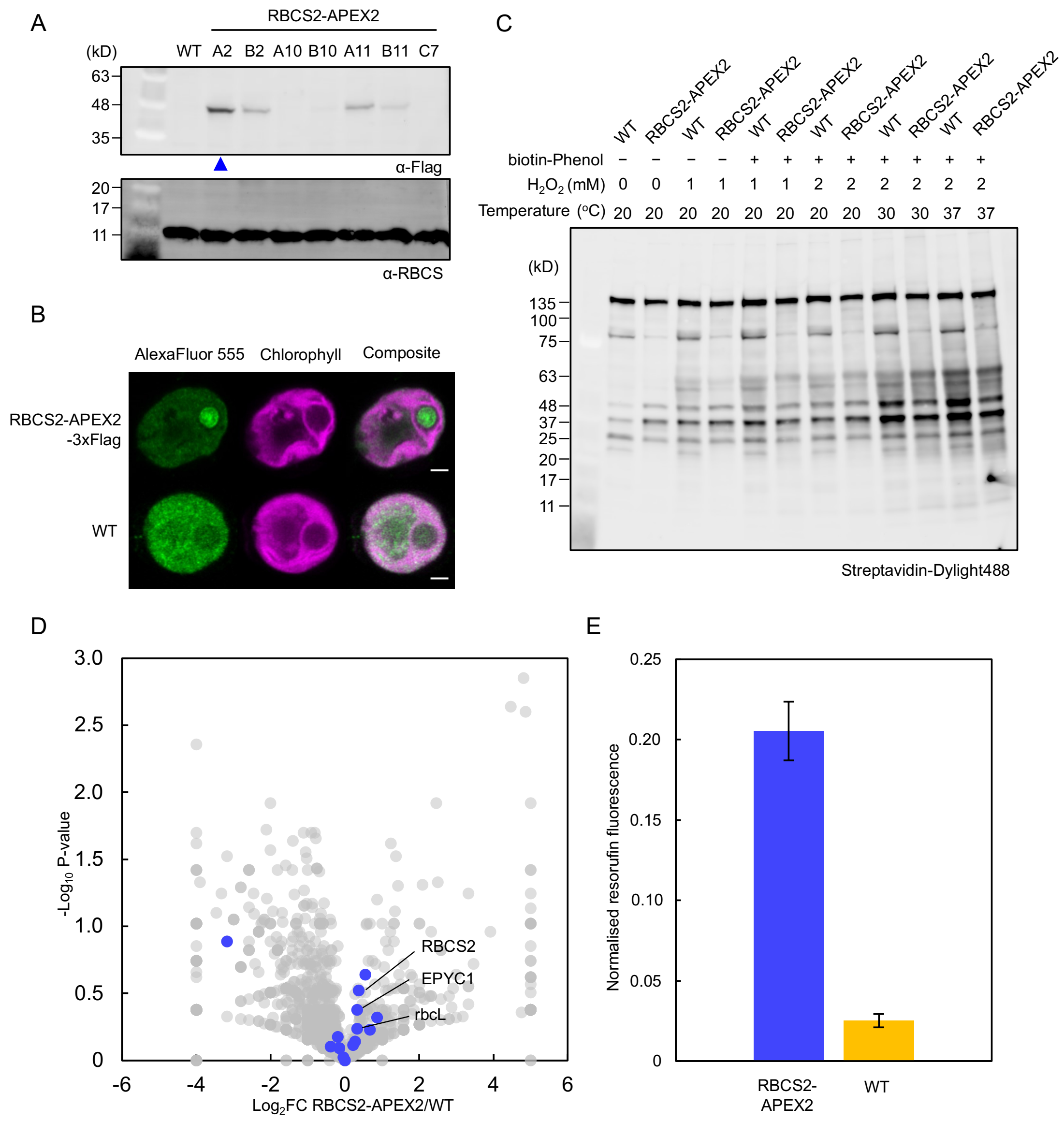

Supplemental Figure S1. APEX2 does not efficiently label pyrenoid proteins in the Chlamydomonas chloroplast. (Supports Figure 1.) **A,** Abundance of RBCS2-APEX2 in Chlamydomonas CC-4533 transformants was verified by immunoblotting whole cell lysates with anti-Flag antibody. Anti-RBCS was used as a loading control. Blue arrow denotes the strain chosen for later labeling experiments. **B,** Localization of the RBCS2-APEX2 fusion protein as determined by immunofluorescence using anti-Flag antibody. Green and Magenta signals denote Alexa Fluor 555 and chlorophyll fluorescence, respectively. Non-specific binding of the anti-Flag antibody results in background signal in WT cells. Scale bars, 2 µm. **C,** Labeling efficiency of RBCS2-APEX2 was tested by incubating expressing strains with 2.5 mM biotin-phenol substrate for 2 h. H_2O_2 activator was added at different concentrations (0–2 mM) and activation was carried out at a range of temperatures (20–37°C). Biotin labeling was visualized by immunoblotting whole cell lysates against streptavidin. **D,** Volcano plot representing the Log_2 fold-change of spectral counts from RBCS2-APEX2 compared to untagged WT labeling experiments. Gray and dark blue dots represent detected proteins and known pyrenoid proteins, respectively. Significance was determined via t-test. Proteins detected only in WT are set to –4 Log_2 FC and proteins only detected in RBCS2-APEX2 are set to 5 Log_2 FC. **E,** Amplex-red Assay was carried out to determine the peroxidase activity of RBCS2-APEX2 strains. Untagged WT and RBCS2-APEX2 strains were incubated with Amplex-red reagent and activated with H_2O_2 at 1 mM. Resorufin fluorescence emission (excitation: 535–555 nm; emission: 580–620 nm) was normalized against chlorophyll autofluorescence (excitation: 610–630 nm; emission: 660–695 nm) (n=6). Error bars indicate standard error.

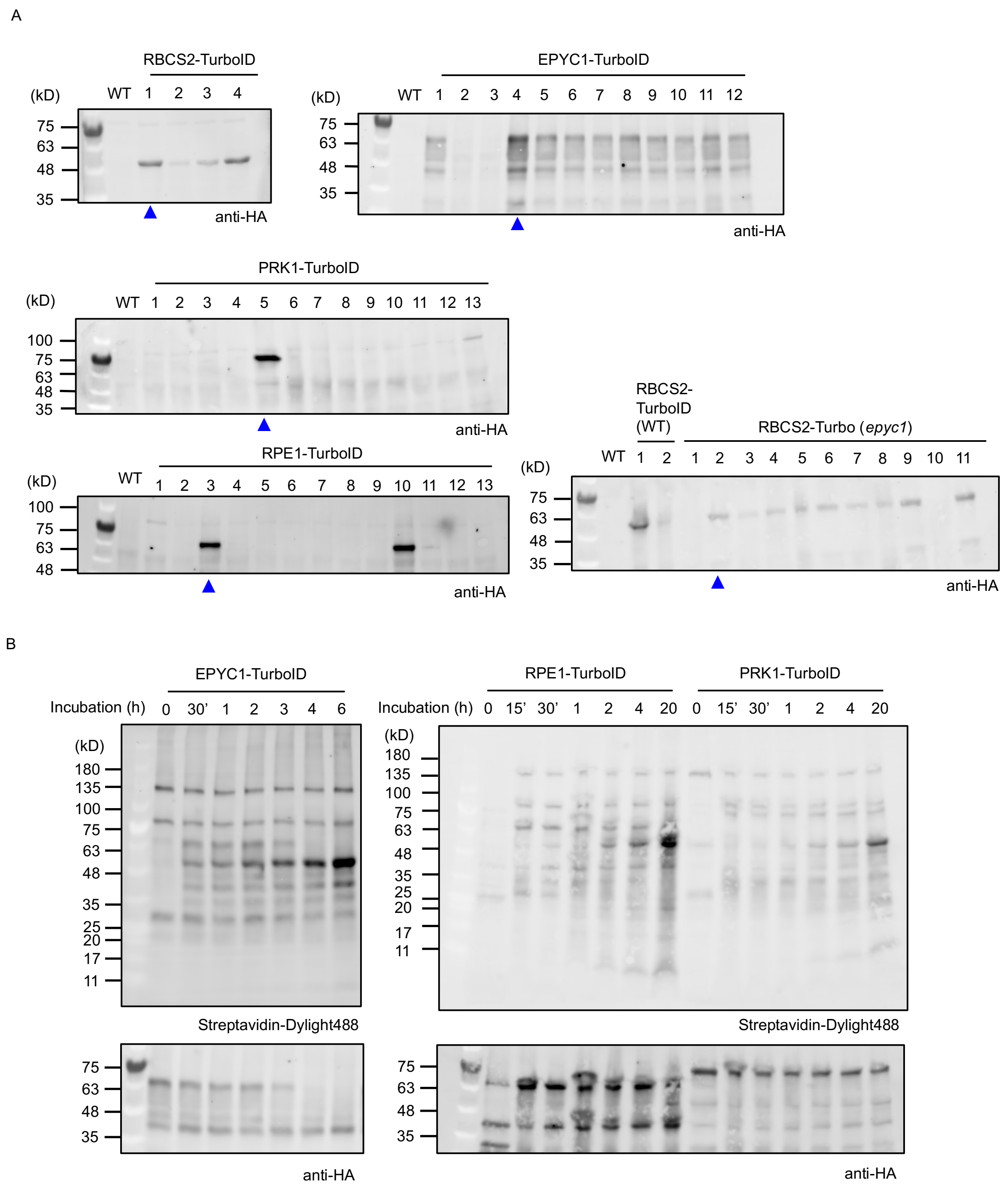

Supplemental Figure S2. Screening strains for TurboID accumulation and activity. (Supports Figure 1, 2, 3 and 5.) **A**, Chlamydomonas cells transformed with *TurboID-tag* plasmids were grown in TAP medium. Protein production was assessed via immunoblotting whole cell lysates from picked strains with anti-HA antibody. Blue arrows denote the strains used in labeling experiments in Figures 1, 2, 3 and 5. **B**, Labeling activity of the various TurboID-tagged lines was assessed by incubating them in 2.5 mM biotin for a range of durations (0–20 h). Anti-HA antibody was used to assess protein accumulation.

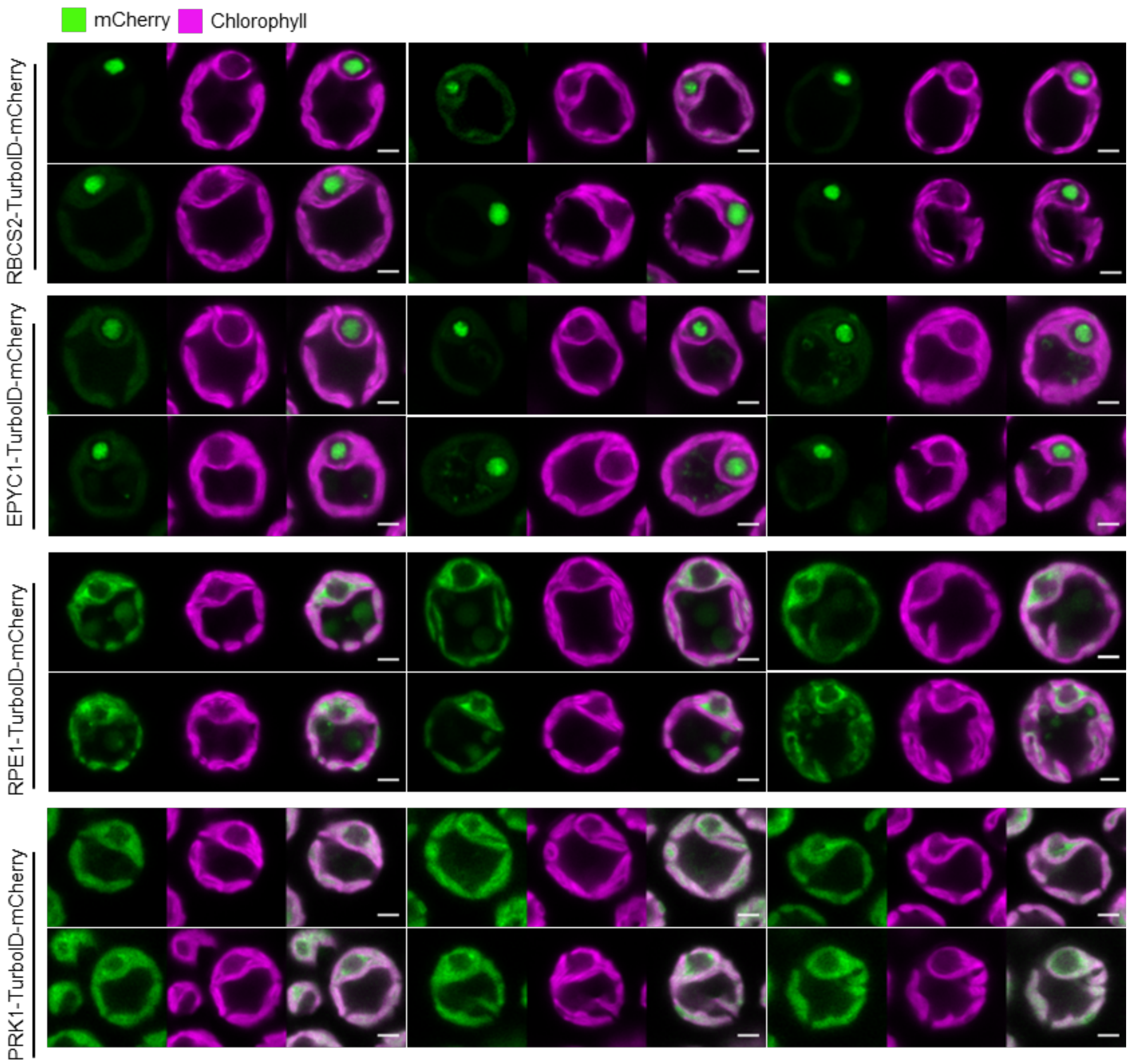

Supplemental Figure S3. Confocal imaging of mCherry tagged TurboID strains. (Supports Figure 1 and 3.) *RBCS2*, *EPYC1*, *RPE1* and *PRK1* were cloned in-frame with a *TurboID-mCherry* sequence at their 3’ ends. Images shown are representative images from two independently transformed strains. Green and magenta signals denote mCherry fluorescence and autofluorescence from chlorophyll, respectively. Scale bars, 2 µm.

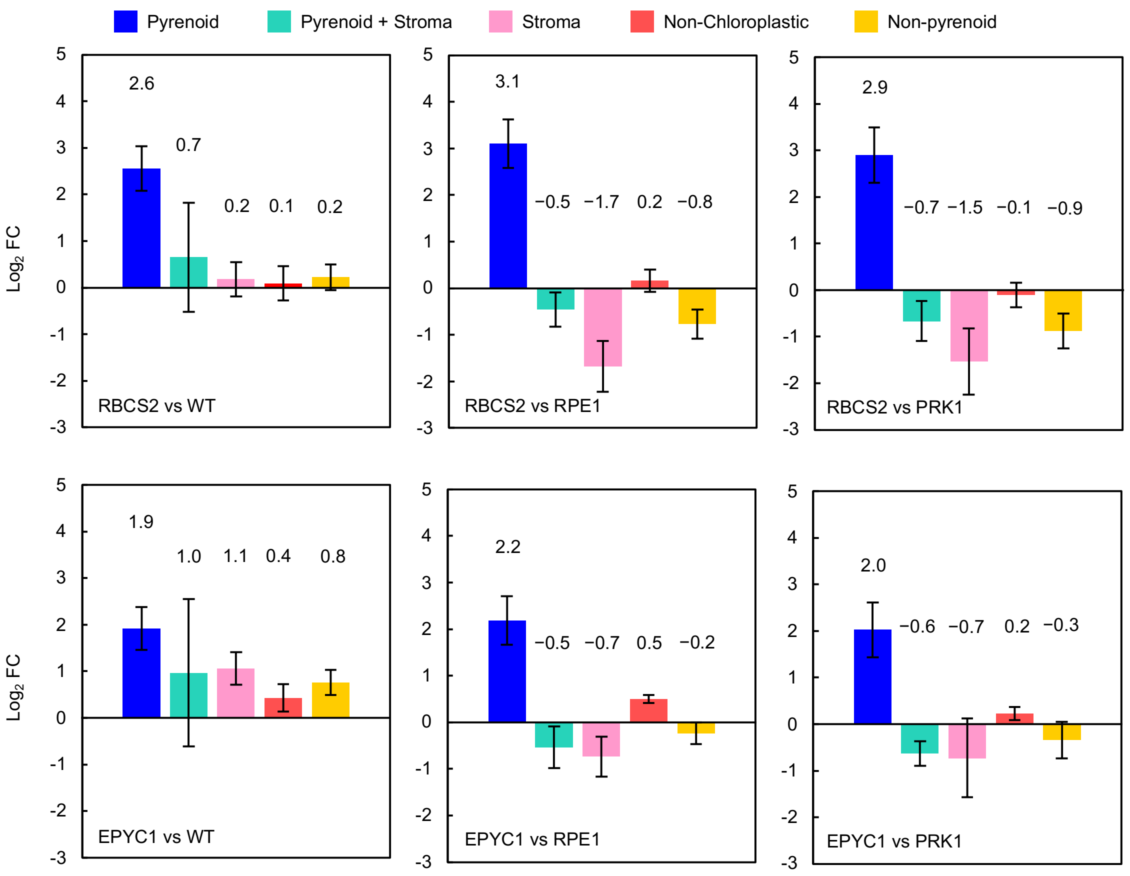

Supplemental Figure S4. Enrichment of differentially localized proteins using different controls. (Supports Figure 3.) Averaged Log_2FC for each comparison group was calculated according to their localization classification, a final category "non-pyrenoid" was created by combining all the non-pyrenoid proteins. Pyrenoids (n=16-19), Pyrenoid+Stroma (n=3-5), Stroma (n=9-13) and Non-chloroplastic (n=10), non-pyrenoid (n=23-27). The benchmark proteins used here are shown in Supplemental Data Set S3. Error bars indicate standard error.

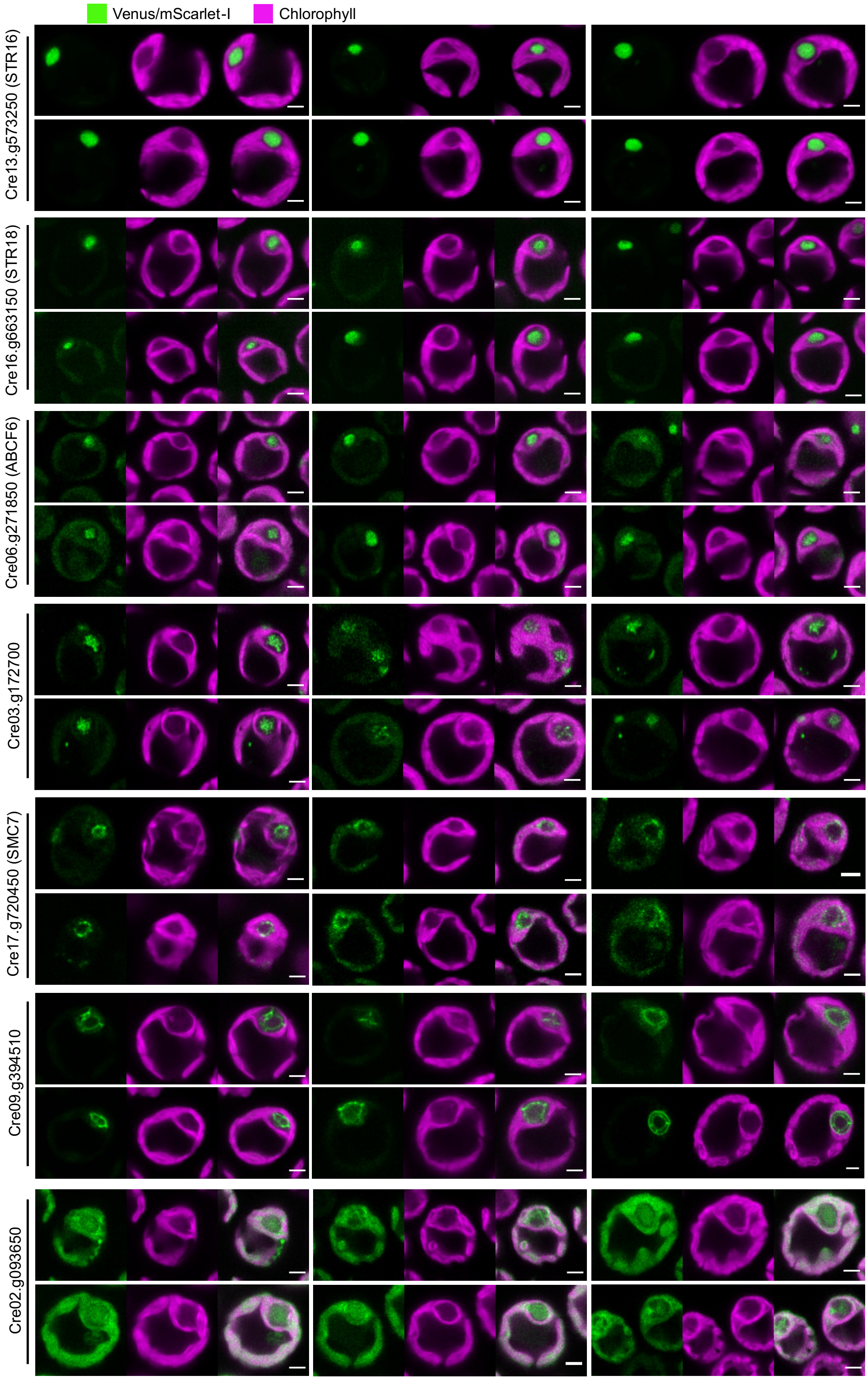

Supplemental Figure S5. Additional images of the TurboID identified pyrenoid proteins. (Supports Figure 4.) Two independently transformed strains were imaged for each gene; representative images shown here. Green and magenta signals denote Venus or Scarlet fluorescence and chlorophyll autofluorescence, respectively. Scale bars, 2 μm.

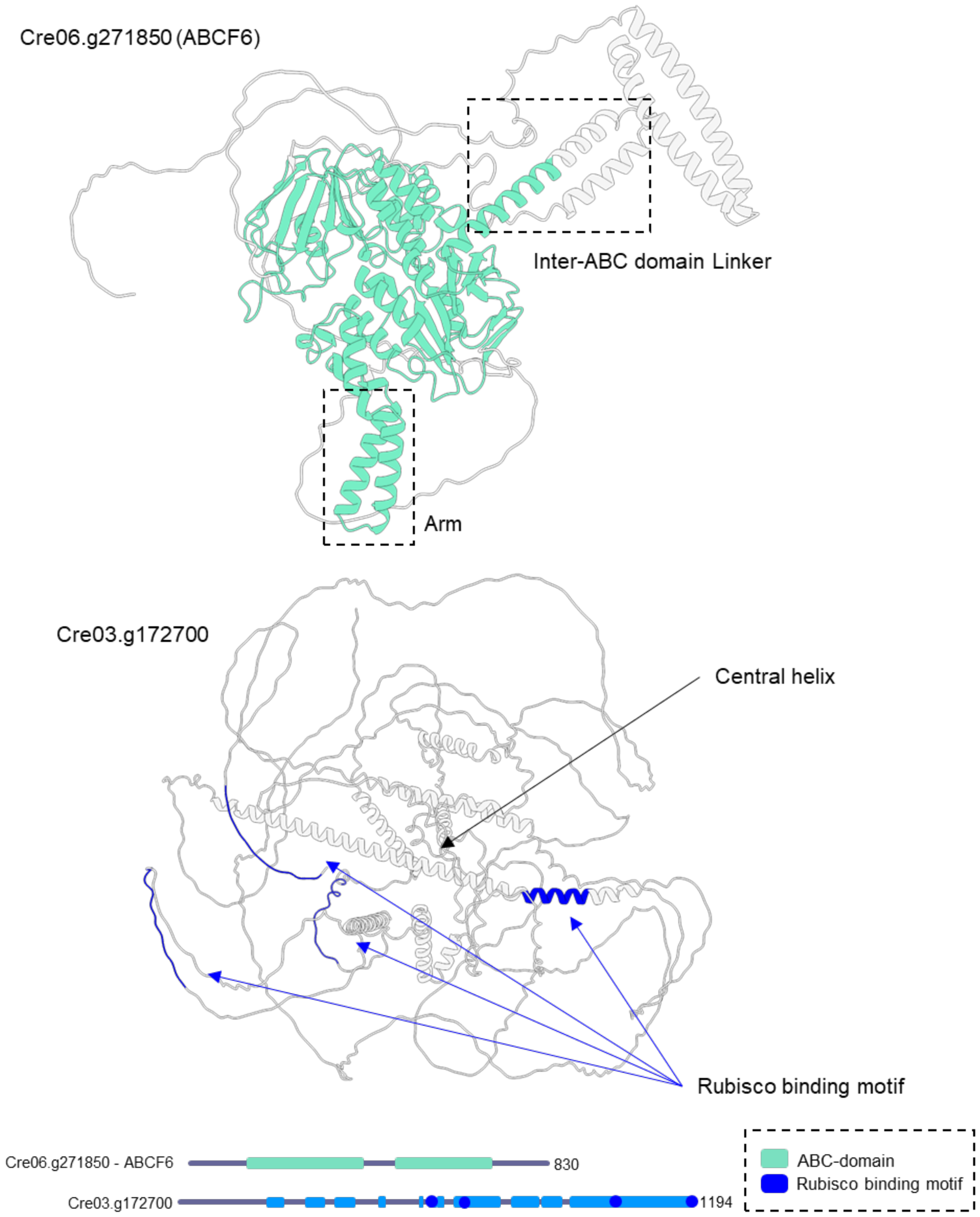

Supplemental Figure S6. AlphaFold-modeled structures for the proteins encoded by Cre06.g271850 (ABCF6) and Cre03.g172700. (Supports Figure 4.) The protein models (A0A2K3DX68 for Cre03.g172700 and A0A2K3DNE2 for Cre06.g271850) were obtained from the Uniparc archive (UniProt Consortium, 2021; Jumper et al., 2021). Dashed rectangles on ABCF6 denotes either the inter-ABC domain linker commonly found on antibiotic resistance-conferring ABCF protein where the linker is hypothesized to interacts with Ribosome-bound antibiotics (Murina et al., 2019) or the L1 ribosome-binding arms. The four Rubisco binding motifs on Cre03.g172700 are colored in blue (blue arrows). The black arrow denotes the central alpha helix. Schematic diagrams and color scheme of the two proteins from Figure 4B is presented below.

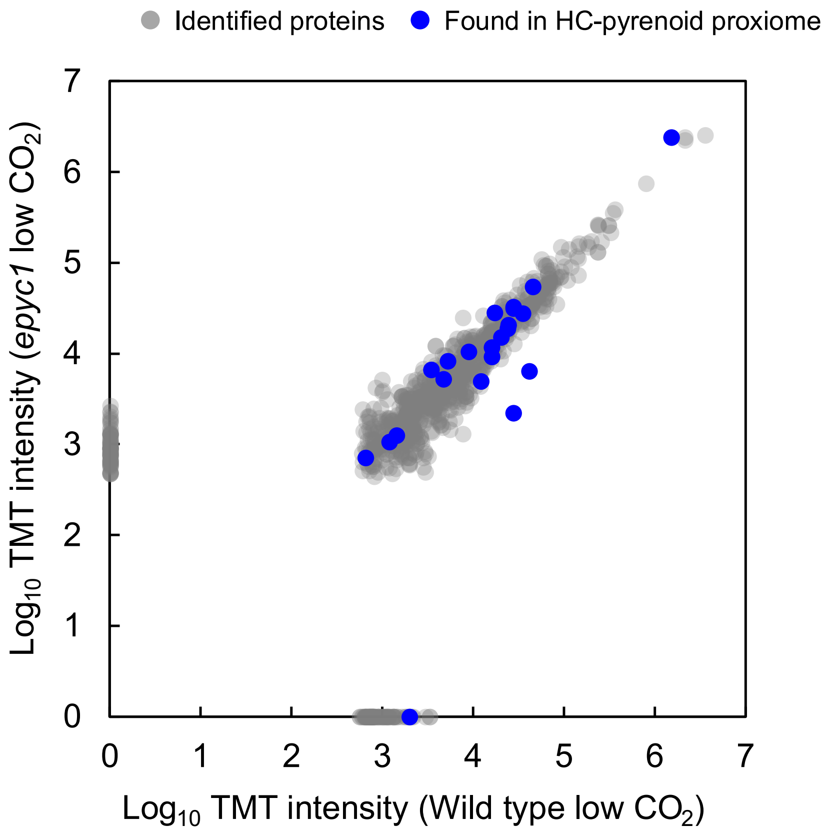

Supplemental Figure S7. Comparison of protein abundance between wild-type and *epyc1* background strains expressing *RBCS2-TurboID* after streptavidin-affinity purification. (Supports Figure 5.) The Log-transformed TMT intensity values of the normalized intensity from wild type and *epyc1* grown in low CO_2 are compared. Comparison between the two background strains suggests there are only minor difference between the overall protein profile.
