# Supplementary material for: A phase-separated CO2-fixing pyrenoid proteome determined by TurboID in Chlamydomonas reinhardtii
Source: Plant Cell. 2023 May 17;35(9):3260–79. doi: 10.1093/plcell/koad131 (PMC10473203; doi:10.1093/plcell/koad131)
Supplement: koad131_Supplementary_Data [file tpc22.01174Peer_Review_Report.pdf › tpc22.01174Peer_Review_Report.pdf]

The phase separated CO_2-fixing pyrenoid proteome determined by TurboID

Chun Sing Lau; Adam Dowle; Gavin H. Thomas; Philipp Girr; Luke CM Mackinder

Corresponding author: *Luke CM Mackinder*, *luke.mackinder@york.ac.uk*

Review timeline:

**TPC2022-BR-01174**	Submission received:	Dec. 1, 2022
	1st Decision:	Feb. 2, 2023 *revision requested*
**TPC2022-BR-01174R1**	1st Revision received:	April 6, 2023
	2nd Decision:	April 7, 2023 *acceptance pending, sent to science editor*
	Final acceptance:	May 8, 2023

REPORT: (The report shows the major requests for revision and author responses. Minor comments for revision and miscellaneous correspondence are not included. The original format may not be reflected in this compilation, but the reviewer comments and author responses are not edited, except to correct minor typographical or spelling errors that could be a source of ambiguity.)

TPC2022-BR-01174 1st Editorial decision – *revision requested* **Feb. 2, 2023**

We have received reviews of your manuscript entitled "The phase separated CO2-fixing pyrenoid proteome determined by TurboID." Thank you for submitting your best work to The Plant Cell. The editorial board agrees that the work you describe is substantive, falls within the scope of the journal, and may become acceptable for publication, pending revision and potential re-review.

We ask you to pay attention to the many very specific and constructive suggestions made by the reviewers. No additional experiments will be required.

-- Reviewer comments:

[Reviewer comments shown below along with author responses]

TPC2022-BR-01174R1 1st Revision received **April 6, 2023**

Reviewer comments and **author responses**:

Reviewer #1:

The manuscript by Lau et al. utilized proximity labeling to investigate the protein components of pyrenoid in Chlamydomonas, a new model organism in which TurboID is applied. The manuscript was clearly written, and the data provided support for the successful identification of at least part of the pyrenoid proteome in Chlamydomonas. I have some suggestions that could further improve the manuscript before its publication.

We would like to thank Reviewer #1 for their positive comments and constructive feedback.

I have a different opinion on the conclusion drawn from Fig. 2E and 2F, where the authors state that "chloroplast localized proteins exhibit an increase in labeling from 1 to 4 hours while non-chloroplastic protein remains stable". Except for the time point of 2h (statistics needed), the difference appears to be systematic and localization-independent. Therefore, the concern of substrate labeling by non- pyrenoid-localized RBCS2 with equal efficiency remains, and the discussion in lines 222-232 shall be revised. Is it true that all RBCS2 localize to pyrenoid under the photoautotrophically condition used here?

We thank the reviewer for this comment and agree that temporal changes in labeling appear to be partially systematic, particularly in the chloroplast localized groups (P, PS and S). Our main objective with Fig 2E and F was to point out that RBCS2-TurboID driven biotinylation preferentially enriches known pyrenoid and chloroplast

localized proteins, as evidenced by the consistently higher enrichment seen in chloroplast proteins regardless of whether they fall within the pyrenoid. To explore if any temporal labeling trends were significant we carried out a two-way repeated measures ANOVA analysis to explore the difference between protein labeling at the range of timepoints. We found that the increase in biotinylation is statistically significant only at the 4 hour point for pyrenoid proteins and stromal proteins, while non-chloroplastic proteins showed a statistically significant decrease at 2 hour and 8 hour time points (see L219-221). Additionally, the increase in labeling from 1 to 4 hours is more pronounced in pyrenoid proteins than in stromal proteins. Since approximately 90% of Rubisco is concentrated to the pyrenoid in the low CO2 condition our *Chlamydomonas* strain is cultivated in (Borkhsenious et al., 1998; Mackinder et al. 2016), we do expect a fraction of non-pyrenoid-localized RBCS-TurboID. This could explain the systematic temporal increase between 1-4 hours of pyrenoid+stroma and stroma proteins. We did touch on this from a phase separation perspective (L231-242) but have now referenced the relevant experimental studies that show even when the CCM is fully induced a fraction of Rubisco is found outside of the pyrenoid (L242-245). The ANOVA analysis has been added on to Figure 2E, where test statistics are included in the new Supplemental data set 8.

Technical concerns and suggestions for Figure 3:

1) Although protein biotinylation is inducible in the EPYC-TurboID line (Fig. 3B), I am not sure whether EPYC1 is well-expressed. Please indicate the EPYC1 band in the HA blot and self-biotinylated EPYC1 in the streptavidin blot.

   We find that rather than poor expression, the EPYC1-TurboID protein shows signs of degradation in our labeling conditions. Intrinsically disordered proteins commonly show a propensity to degrade. In fact, similar degradation of the EPYC1 protein has previously been reported when expressed in higher plants (Nicky et al., 2020). The strain examined in this study was picked from Strain 4 in the Supplemental Figure 2A, where its activity was also tested (Supplemental Figure 2B). We have now added the anti-HA immunoblot on the EPYC1-TurboID assessment as well (Supplemental Figure 2B). There we found that the self biotinylation signal diminishes with extended incubation time, however the overall biotinylation signal still increases without the appearance of additional bands suggesting that the biotinylated target proteins are not changing. We therefore reasoned that the overall biotinylated proteome is unperturbed. We have now included (L297-300) "While we saw evidence for EPYC1-TurboID protein degradation with increased biotin incubation time, the overall biotinylation signal does not appear to be perturbed (Supplemental Figure 2B). We therefore continued using EPYC1-TurboID with a 4 hour biotin incubation time in our MS experiments."

2) Another concern that I have is the use of RPE1 as a non-pyrenoid control because the protein seems to be concentrated at the periphery of the pyrenoid (Fig. 3A), which may label pyrenoid contents and result in potential false negatives.

   RPE1 localization external to the pyrenoid has been previously demonstrated by fluorescent protein tagging and biochemical fractionation, and is in line with the other calvin cycle enzymes such as FBA3, GAP3 and SBP1 (Kuken et al., 2018). We agree that RPE1 localization shows enrichment at the pyrenoid periphery, however we reasoned that the starch sheath barrier surrounding pyrenoid and thought to separate RPE1-TurboID from the pyrenoid matrix will limit biotin radical penetration into the matrix. Additionally, we reason that the enrichment of RPE1-TurboID at the pyrenoid periphery is particularly well suited to establish a stringent pyrenoid proxiome by removing non-matrix pyrenoid periphery proteins. In the new Supplemental Figure 3 we have now included additional images of all TurboID-mCherry fusions supporting the absence of PRK1 and RPE1 from the pyrenoid matrix.

3) I suggest labeling all candidates that pass the threshold (e.g. logFC>1 and p<0.05) in Fig. 3F. compared to both controls. This ratio matric analysis shall yield a high-confident candidate pool for the bait. With this high confidence proxiome, the authors can also compare RBCS2 and EPYC1 proxiomes to strengthen their argument that these two baits label the same group of proteins in pyrenoid.

   We agree with the reviewer that using these parameters can yield a high-confidence candidate pool for the pyrenoid baits. Interestingly, the ROC analysis we employed to generate the enrichment threshold yields a value very close to Log2FC = 1, which is 1.31 for RBCS2/RPE1 and 1.14 for RBCS2/PRK1. Once proteins that do not show a statistically significant difference are removed, the resulting candidate pool is very similar to the ones generated by ROC-thresholds (we have showed these proteins in Fig. 3F as colored circles). This pool is already contained within the candidate pool we have now termed the "high-confidence pyrenoid proxiome". When analyzing the RBCS2 and EPYC1 proxiomes obtained this way, we found that they are remarkably similar where 12/15 of proteins found in the

EPYC1 proxiome can also be found in the RBCS2 proxiome. We have added a sentence on L390 "With 12 out of 15 proteins identified in the EPYC1-TurboID vs stromal controls (RPE1 and PRK1) are also found in the RBCS2-TurboID vs stromal controls analysis (Figure 3D and Supplemental Data Set 5)." This strengthens our argument that both pyrenoid baits generate comparable labeling.

4) In Fig. 3D, I am not sure if it is appropriate to name the 84 identified proteins "pyrenoid proxitome" while the 30 preys as "pyrenoid matrix proteome". To this reviewer, the latter could be simply a candidate pool with higher confidence than the prior due to more precise controls. Analysis that compares the 84 and 30 proteins is needed.

We have renamed the pyrenoid matrix proxiome to "high-confidence pyrenoid proxiome (HC-pyrenoid proxiome)". Our reasoning behind the original naming stems from the observation that pyrenoid periphery enriched proteins such as the LCIB and SBE3 are found in the "pyrenoid proxiome" but not in the "matrix proxiome". Indeed, we agree with the reviewer that instead this could similarly be interpreted as a higher confidence list. We further compared the two proxiome datasets and found that the matrix proxiome retained 11/14 of the known pyrenoid proteins found in the "pyrenoid proxiome". There was also a higher representation of proteins containing a Rubisco binding motif 13/30 (43%) vs 28/84 (28.5%). Consistent with our observation, proteins involved in starch synthesis or proteins that contain a carbohydrate binding domain such as SBE3/STA3/Cre09.g415600 were removed from the matrix proxiome, possibly due to their likely pyrenoid periphery localization. Many chloroplast encoded translation related proteins (ribosomal proteins/RNA polymerase) are also removed. The comparison between the two datasets has also been added on L343-351.

To better support the quality and get more insight from their proximity labeling proteomics, I strongly recommend the authors perform more comparative analyses between the RBCS2/EPYC1 proxiomes and the previously reported pyrenoid MS data by the author and the Jonikas group.

We agree with the reviewer that comparative analysis between previously reported pyrenoid MS data would give us more insight into our proxiome data. We have now performed this comparative analysis (Fig. 3G; discussed L351-360) by comparing our pyrenoid proxiome against all proteins identified as having a significant interaction with RBCS1/2 and/or EPYC1 by AP-MS experiments from Mackinder et al., 2017, as well as the 190 pyrenoid proteins determined by pyrenoid biochemical fractionation and MS from Zhang et al., 2018. We found that 24/84 proteins discovered in our proxiome and 16/30 protein in our HC-pyrenoid proxiome overlap with at least one of the previously determined pyrenoid "proteomes". A total of 7 proteins occurred in all three data sets, 5 of which were known pyrenoid proteins and the other two confirmed in Figure 4. We further investigated the RBM representation in all three datasets, and found that the distributions were similar across all three works. This comparison further demonstrates the robustness of our proximity labeling approach and also the complementarity of the different methods.

A few questions about data shown in Figures 4 and 5:

Fig.4: Could the authors provide more information on how the 8 proteins were chosen to validate pyrenoid localization, and are they part of the 30 high-confidence candidates tested? Has the localization of all the 30 high-confidence candidates been tested? Why was STR16 tested as this protein does not even pass the threshold?

Selection of previously unlocalized proteins for confirmation was based on several factors. The primary basis was from preliminary proximity labeling experiments during the establishment of the methodology and prior to having all of our robust controls in place. Although STR16 was below our enrichment threshold in early experiments (as highlighted in Figure 4A) it was in the HC-pyrenoid proxiome once we had established additional controls.

We have not established localization of all the 30 high-confidence candidates. A significant proportion of these (11) have already been shown to be pyrenoid localized by our lab or our collaborators (see Meyer et al. 2020, Mackinder et al. 2017). In this study we localize two of them to the pyrenoid: STR16 and STR18 (Figure 4). We have not tried to localize the outstanding targets but think these should be of a high priority for the field to target.

Fig.5: It looks like RBCS2-TurboID in the epyc1 background could serve as a good control for the identification of pyrenoid protein. Have the authors performed this analysis? If so, what is the pyrenoid proxiome compared to the one obtained using RPE1 and PRK1 as control?

We agree with the reviewer, and explored if this dataset could be a good control for identifying pyrenoid proteins. We have compared the proteins labeled by RBCS2-TurboID in WT against RBCS- TurboID::*epyc1* to the HC-pyrenoid

proxiome which is represented in Figure 5C. We found that a considerable number of pyrenoid proteins detected in the MS analysis were found to be present in both data sets. However, 11 out of 25 proteins within the high-confidence pyrenoid proxiome are not enriched in the RBCS2-TurboID in the *epyc1* background - indicating that the RPE1/PRK1 controls give a broader proxiome. We hypothesize that these differences stem from the residual pyrenoid that is present in the *epyc1* mutant, which is thought to be due to a fraction of Rubisco being recruited to pyrenoid-tubule thylakoid membranes by pyrenoid proteins that interact with membranes.

Reviewer #2:

In this study, Lau et al. established proximity labeling technique to define "proxiome" in pyrenoid of Chlamydomonas chloroplasts, which exists as liquid-liquid phase separation (LLPS).

As authors stated, by using conventional affinity purification mass spectrometry, it would be very difficult to precisely determine the proteome (or proxiome) of pyrenoid, due to its sensitivity to changing environments. This reviewer agrees with authors that the application of proximity labeling method such as TurboID can be a good way to resolve this type of obstacle.

One prominent strength in this study is that authors succeeded in the validation of developed methods through the identification of previously well-known interacting partners in Chlamydomonas pyrenoid.

In addition, authors identified (previously unknown) novel pyrenoid proteins, which will be the basis for further in-depth characterization of algal pyrenoids and application of algal CCM to C3 plants.

Furthermore, authors attempted to address dynamic pyrenoid proteome in the context of CO2 concentrations and phase separation, thereby further validating TurboID method established in this study.

We would like to thank the reviewer for their positive comments on the manuscript. This reviewer has one minor comment.

In Figure 1, the results regarding self-biotinylation of RBCS2-TurboID (or biotinylation of Rubisco large subunit) in the presence of exogenous biotin are convincing.

However, why were the band intensities in anti-HA blots increased in the presence of exogenous biotin? (Especially in Figure 1D). Moreover, in Figure 1B, the anti-tubulin blot is of poor quality, and thus requires further improvement.

In Figure 1B, we have repeated the immunoblotting using the same protein extracts which improves the quality of alpha-tubulin signals, which replaces the original panel. For Figure 1D, we agree there is a slight increase in intensity for the first four RBCS2-TurboID lanes but it then plateaus. We think this is due to a minor variation in protein transfer across the blot but that this does not alter any of our conclusions or the interpretation of the data. We also do not see any variation upon biotin addition in Figure 3B or 4A, again supporting that HA intensity of RBCS2-TurboID does not increase upon biotin addition.

Reviewer #3:

In this manuscript the authors aimed to develop a method to identify the pyrenoid "proxiome" - to identify putative Chlamydomonas pyrenoid components. Two proximity labelling approaches were trialed - APERX2 and TurboID, using two different known essential pyrenoid components as baits. Both fusion proteins localized to the pyrenoid. While it appears APEX2 was functional within the cell, proteins were poorly biotinylated. TurboID, on the other hand, biotinylated hard, with the likely explanation being differences in Chlamydomonas' permeability to the biotin-phenol and biotin substrates for APEX2 and TurboID, respectively. Substrate concentration and labelling time were optimized for TurboID fusions, followed by substantial further optimisation for optimal labelling conditions with respect to protein identification via mass spec by tracking selected benchmark proteins (with known sub-cellular localization). These were compared to background levels of biotinylation in WT Chlamydomonas. Further noise was removed by comparisons of mass spec data with protein sets representing various cellular sub-compartments. Using this method

the authors identified novel putative pyrenoid components, and a first pass validation was performed by looking at localization of these proteins to the pyrenoid in Chlamydomonas.

Overall, this is a decently written text, and really great data. Concepts and results are generally well communicated. Discussion about potential functions of the proteins they identified could be either further developed or made more clear. I suggest the following minor revisions to improve communication. Although the list is long, I don't envision it would require major effort to address them.

We would like to thank reviewer #3 for their extensive feedback and suggestions that we think have considerably improved the manuscript.

General minor grammar

-In general, need a comma before the word "which"

-"Data" is plural - therefore - "these data"

-Relevant hyphens would improve readability - e.g. "matrix-generated", "oxygen-sensitive" "rhodanese domain-containing proteins" etc.

-Check singular/plural and prepositions throughout text.

-Extra attention to language in methods and figure legends.

We have carefully checked the text and made the above suggestions where we deemed appropriate. All changes are tracked.

Specific feedback:

1. Lines 104-107. Clarify sentence. Current construction makes it sound like APEX2 and TurboID were fused to the same RBCS2 (a RBCS2-APEX2-TurboID fusion)

L104-105 amended to "Either the APEX2 or the TurboID tag were fused to the …"

2. Line 115. Consider referencing Supp Fig 1A after mentioning A2.

Reference to Supplemental Figure 1A has been added.

3. Line 192. Incubated/incubation used twice in same sentence.

L199: has been amended to " …the untagged WT lines were incubated with 2.5 mM biotin across a range of time points…."

4. Lines 217-218. Based on your data, 4 hours is certainly an acceptable time point to choose for further experiments, but can you really claim that it has likely approached full saturation without finer time sampling? For example, 5 h could have double the signal from 4 h. (Not requesting additional experiments.)

We agree with the reviewer that we might have been overzealous to state that we have achieved full labeling saturation within 4 hours as we have not tested further time points between 4-8 hours.

However, the labeling increase of pyrenoid proteins at 8 hours was a much smaller increase compared to the transition of 2 to 4 hours. Considering the biotinylation signal on whole-cell lysate (Figure 2E), we believed that the labeling is indeed approaching saturation. To more accurately describe this, we have amended L224-7) to "While further testing on finer time points will be required to establish the true saturation point in biotin labeling, our data suggests that protein labeling by RBCS2-TurboID begins to approach saturation around 4 hours."

5. Line 262. Something is off with this heading. Perhaps changing "enables" to "enable" will rectify?

L284: "enables" has been amended to "enable"

6. Line 301. Rewrite sentence to remove "we wondered"

L326: This has now changed to "Next, we set out to see if comparison against stromal control lines…"

7. Lines 301-302. Where was this hypothesised?

We have now deleted "as hypothesized" (L328).

8. Line 308. Ie i.e.

Amended

9. Line 325. Replace the word "argue." The data doesn't argue.

L368: Changed to "This similarity suggests …"

10. Line 333. Consider replacing "result in" with "generates" (or similar)

L376: Amended to "generate comparable…"

11. Lines 334-335. Are the two clauses in this sentence repeating each other? i.e. if RBCS and EPYC1 are both essential for phase separation and pyrenoid formation - does this not make them inherently the "two core proteins of the pyrenoid"?

We think the two clauses here represent different properties of Rubisco and EPYC1. Rubisco and EPYC1 are highly abundant proteins within the pyrenoid, however this abundance does not imply their properties of being essential to phase separation. To better communicate this, we have amended this sentence to (L377-379): "Rubisco and EPYC1 are the two major components of the pyrenoid. Their interactions with each other are both essential for phase separation and pyrenoid formation."

12. Line 340. Use "more broad" or "broader" (not "more broader"). Suggested rewrite for this sentence: "... Rubisco interactors or has captured the broader pyrenoid proteome."

L384: Amended according to suggestion.

13. Line 347. Is it possible to show the line and R2 in Fig. 3H for known pyrenoid proteins?

This has been amended, regression and R^2 value is now shown on Figure 3I (previously Figure 3H).

14. Line 368 onwards. Revisit this text - what is the point of it? Why do we want to hear about it? (we really do want to hear about it!). Make sure that comes across. Make it clearer what information you are getting out of alphafold. Make explicit the connection/analogy between the [RBM-disorder]-... domains in Cre03.g172700 and other things that interact with Rubisco in the pyrenoid (e.g. EPYC1), if this is indeed something you are trying to infer in the text.

Our goal here is to describe the diverse localization patterns we observed and the presence of certain predicted protein domains to suggest some possible function for these pyrenoid proteins. To that end, we have added more clarity to our text here by stating explicitly our hypothesized role of these identified proteins. We additionally removed AlphaFold predicted structures of STR16, STR18, Cre09.g394510, Cre02.g093650 from Supplemental figure 6, since these were not used in the analysis described in the text.

15. Line 371-372 - what does "as opposed to CAS1 and RBMP2 mean? That CAS1 and RBMP2 do not have a rhodanese domain? Why would them not having it be important for the reader to know? Also choose rhodanese or Rhodanese.

CAS1 and RBMP2 also have a rhodanese domain. Here we are referring to the lack of a catalytically important cysteine in the CAS1 and RBMP2 protein. In contrast, STR16 and STR18 contain this cysteine in their active site and are most-likely catalytically active. To improve clarity, we rephrased the sentence accordingly "STR16 and STR18 contain Rhodanese (thiosulfate sulfurtransferase) domain like the previously identified pyrenoid proteins CAS1 and RBMP2. In contrast to STR16 and STR18, CAS1 and RBMP2 lack a critical cysteine in their active site and thus presumably are catalytically inactive"

16. Line 376. Which class of proteins shown in Fig. 3G contain an iron-sulfur cluster domain? Indicate in text or clearly in Figure.

We mistakenly referred to Figure 3G when mentioning iron-sulfur cluster domain, we were in fact referring to the pyrenoid proxiome list instead (Supplemental Data set 5). We have amended L428- 430 to "The latter is particularly interesting as multiple proteins in the pyrenoid proxiome contain an iron-sulfur cluster domain, such as

Cre02.g093650, Cre08.g365692 and Cre15.g643600 (Supplemental Data Set 5)" to indicate what proteins we are referring to here.

17. Line 385. Use different description to "latter half" e.g. the "C-terminal half"

Amended according to the suggestion (L440) "... that its C-terminus…"

18. Line 379-380. Was alphafold necessary to predict the arm and linker? If these (arm and linker) insertions were present in the motifs in Cre06.g271850 (and they are by definition a feature of ABC- Fs?) then alphafold isn't revealing anything new. Perhaps it helps confirm, but alphafold is going to fold anything with that motif the same way because it has been trained to. Could say alphafold output was consistent with expected domains (or similar).

We agree with the reviewer that AlphaFold modeling of ABC-F helps confirm the ABC-F family annotation rather than act as an independent line of evidence here. We have amended line 379 to "The AlphaFold modeling of ABCF6 presents a structure consistent with its ABCF annotation (Supplemental Figure 6; Jumper et al., 2021), where the canonical arm and linker domains are present."

19. Lines 405-407. This is a dense sentence to pull the meaning from. Perhaps the following is clearer? "The localization of Cre03.g158050 specifically to the pyrenoid periphery suggests that there may be sub-pyrenoid spatial control of protein phosphorylation."

As explained above, we have now removed Cre03.g158050 data from the manuscript.

20. Lines 408-410. Reword sentence for clarity. Remove the word "together." Here (or elsewhere in manuscript) explicitly state that you think there is a difference between a protein that is in the pyrenoid and a protein that is required for pyrenoid formation (which is the current vibe from the sentence).

We have amended this to (L469-470): "Collectively, these new pyrenoid proteins represent novel candidates for further investigation into pyrenoid formation and pyrenoid function."

21. Line 458. Kd (subscript d)

It has now been amended.

22. Line 446. And from line 540. Questions about protein content vs enrichment. How do you know that less enrichment is not a result of reduced protein content? For example, are RBM-containing protein pools constant in the presence/absence of a pyrenoid? Or do they reduce in the absence of a pyrenoid?

To address this question, we compared the protein abundance between the wild-type and *epyc1* mutant grown under low CO2 conditions (Supplemental Figure 7). We saw that the majority of proteins have comparable abundance which suggests loss of *epyc1* might not severely impact protein content. This is also in agreement with a previous study which found the loss of *epyc1* exhibit no observable difference in Rubisco content (Mackinder et al., 2016), we therefore suggest that the changes in labeling is primarily based on reduced enrichment. We have added this analysis on L520-522 "The differences seen are unlikely due to changes in protein abundance between WT and *epyc1* as these remained highly comparable (Supplemental Figure 7)."

23. Lines 458-460. Do you mean "pyrenoid formation" (i.e. a process - these proteins are only enriched when pyrenoids are actively in the process of forming) or "presence of pyrenoids" (i.e. these proteins only enriched when they are all concentrated within a pyrenoid).

Here we are referring to the presence of a pyrenoid. We have amended this section (L509-525) to clarify this.

24. Lines 458-460. Does this imply that pyrenoid-localised proteins containing RBMs are recruited to the pyrenoid by some mechanism other than binding to Rubisco in the stroma?

We think that our data indicates that RBM containing proteins may not always be complexed with Rubisco in the dilute phase (stroma) due to the weak affinity of RBMs. However, once Rubisco is condensed into the pyrenoid there is a high local concentration of Rubisco (and hence RBM binding sites) that results in RBM containing proteins being "trapped" in the pyrenoid (i.e., upon RBM unbinding they rapidly bind to another Rubisco molecule).

25. Line 474. Your data didn't identify this, you used the data to identify it.

L540: Amended

26. Lines 473-481. General check of sentence clarity. On line 477 you could write "... proteins indicate that they are all located within..." this helps drive home that all the missing proteins can be justified by technical limitations - biotin penetration into specific sub-compartments.

We have amended this sentence to be clearer and emphasize that the missing proteins are likely a technical limitation of biotin penetration (L543-545).

27. Line 478. Is "sub-domains" an appropriate description here? Compartments / sub-compartments, perhaps?

We agree that sub-compartments might be more appropriate here, line 544 has been amended.

28. Line 503. E. coli (space)

Amended

29. Line 527. Please refrain from starting a sentence with "And"

It has been replaced by "Additionally, ..." (L596).

30. Line 546. "upon Rubisco condensation into the pyrenoid". Same feedback as point 23. This sentence currently reads like you did real time tracking of enrichment while pyrenoids were forming.

We have amended the sentence to " ...how labeling by RBCS2-TurboID differs when Rubisco condensation into the pyrenoid is disrupted."

31. Line 555. "... which have not yet partitioned..."

Amended according to suggestion (L622).

32. Lines 640 - Specify which buffer for desalting column - was it lysis buffer?

Yes, amended

33. Lines 573 - 830 (methods). Normalise conventions used between each section.

-Do all chemicals have correct subscripts? E.g. MgCl2

-Are all units correct? e.g. 50 uL vs 50 µL

-Space between value and unit, except for temperature (e.g. pH 7.4, 50 V, 10 mM and 17,900)

-wild type? WT? (not wildtype)

-mL or ml

-(v/v) or (v:v)

-minutes or minute or min (and never "mins")

-streptavidin capitalized or not? Capital for sure when it is a brand name, but otherwise doesn't matter as long as consistent.

We have checked the main text and methods section and normalized conventions across the manuscript.

34. Line 588. C-terminus (terminal = adjective, terminus = noun)

This has now been amended.

35. Line 610. "Supernatants were used as protein extract" - I do not know what this means.

We have changed this line to "The cell suspensions were lysed by 5 freeze/thaw cycles and centrifuged at x17900 *g* for 10 minutes at 4°C. The resulting supernatants were used as protein samples in later experiments and stored at -70°C if not used immediately."

36. Line 648. Write triethylammonium bicarbonate before the TEAB abbreviation. Related: Lines 663 and 732: just use TEAB.

This has now been amended.

37. Line 749. Remove abbreviation for trifluoracetic acid as you do not use it elsewhere in manuscript.

This has now been amended.

Figures

38. Figure 1.

-Line 140-141. Clarify sentence. This sounds like the RBCS2-APEX2 encoding construct is transformed into Chlammy, then it is transported to the pyrenoid, then once the construct is inside the pyrenoid the RbCS2-APEX2 fusion protein is translated.

This has now been amended to "The RBCS2-TurboID fusion protein is targeted to the pyrenoid matrix, whereupon addition…"

-Panel D. Express biotin concentrations in mM.

This has now been amended.

-Panels D and E. Add additional labels to gels to help the reader interpret more quickly. Suggest in D: RBCS2-"TurboID (4h)", and E: "RBCS2-TurboID (2.5 mM biotin)".

This has now been amended. We have used hours for consistency throughout the figures and text.

-Panel E. "Time (h)" - not "hr"

This has now been amended. We have ensured consistency across all figures and used hours.

-Panel E. write 15 s and 30 s as 0.25 and 0.5

This has now been amended.

-Panels D and E. Why do the intensities look so different on the different gels? If I interpret correctly, the last lane in D should correspond to the 4 h lane in E - why are non-(rbcL and/or RBCS2-APEX2) bands more intense in panel D?

The contrast used was not consistent between blots which resulted in this visual difference, we have now amended the contrast in panel D. The difference at the 4 hours time point is now minimal between the blots.

39. Figure 2. Panel F is somewhat confusing. Why are there two graphs? Were there approx. 1800 enriched proteins, and you split into two graphs for space reasons?

In Figure 2F we compared two metrics with the top 10% enriched proteins at each time point, (1) PredAlgo predicted localization and (2) the known localization as determined from literature. To provide more clarity, we have amended panel F by moving the color scheme within each graph, and labeled them with "PredAlgo prediction" and "Known localizations".

Panels E and F. Replace "Hour" - with either "hours" or "h"

This has been changed to hours.

40. Figure 3. Panel C. Swap the positions of the RBCS2/PRK and EPYC1/RPE1 graphs. This allows quicker/easier comparisons for the reader.

We have now re-arranged Figure 3 to accommodate the new analysis of previously published pyrenoid proteomes. The new arrangement should enable a quicker/easier comparison.

Panel F. Do you get the same or similar result when plot EPYC1/PRK against EPYC1/RPE1? Comparing EPYC1/PRK1 against EPYC1/RPE1 generates comparable results to what is observed in panel F.

The R^2 value was calculated at 0.58. This comparison has been added to Figure 3F to highlight their similarity.

41. Figure 4—What do the numbers beside the schematics refer to? Amino acid length? Indicate this in Figure and/or legend.

The numbers refer to the amino acid length, this has now been added to the figure.

Supplemental data

42. Supp Fig S3. Make it clear which proteins are in each of the protein sets, e.g. there are more than 18, 10, 15 and 13 proteins listed in Fig S3 for P, PS, S and NC, respectively - which were selected as the benchmarks?

Now Supplemental Figure 4. We initially curated a list of benchmark proteins with known localization via either fluorescence protein tagging or immunofluorescence (Supplemental Data Set 3). This list was then used in all six MS data analyses. However, the benchmark proteins were not consistently detected in all MS experiments, resulting in slight differences in the number of proteins in each class presented in Supplemental Figure 4. We have amended the figure legends of Figure 2 and Supplemental Figure 4 to highlight that the benchmark proteins used are shown in Supplemental Data Set 3".

Supplemental figures

43. Supp Fig 1 and 2. Change colour of yellow arrow - poor contrast in some images. Adjust text in legends accordingly.

Done

44. Supp Fig 1B. Explain somewhere why get diffuse green signal throughout WT.

We have added "Non-specific binding of the Flag antibody results in background signal in WT cells." to the Figure legend.

Supp Fig 1B. Change (hr) to (h)

This has now been amended to hours to be consistent with previous figures.

45. Supp Fig 4. Overall, it is unclear what this figure is adding to the manuscript. Much of the discussion in main text (approx. line 368 onwards) could be made using the PSI-pred data.

(Except perhaps for (i) the tenuous argument made for ABCF1 being an ABCF because of alphafold and (ii) for Cre03.g172700 - but it is not explicitly stated why this is kind of interesting in the main text)). Clearer labelling and presentation of the figure could help make it worthwhile:

-Put gene names in figure, where appropriate e.g. Cre13.g573250 (STR16)

-Indicate N- and C-terminus for each predicted structure (as you describe things relative to these positions in the main text)

-I presume the final version will have higher resolution images - not much can be seen in these images.

-Consider including the schematics from Fig 4B in this figure also (under each structure)

-Label any domains/features that you discuss in the main text: arm + linker domain in ABCF, central helix in Cre03.g172700 and so on.

-Legend - change "Carbohydrate binding" to "Starch binding" to be consistent with Fig 4.

-Rubisco binding motif - really hard to distinguish RBM from Phosphatase and Ferredoxin in the structures. This might be fine in higher resolution images. If not, change the colour scheme. Even when comparing Fig 4 side-by-side with this figure, I cannot see the RBM in the predicted Cre09.g394510 structure.

-Provide the journal with co-ordinates and stats for these predicted structures.

We agree with the reviewer that AlphaFold structures for all the proteins do not contribute to the main text. Since only the analysis of ABCF6 and Cre03.g172700 directly uses the prediction from AlphaFold models, we have removed the other protein predictions. We have additionally labeled the domains that are referred to in the main text

to provide more clarity.

General questions for discussion

46. How would you confirm that any of these new proteins have a role in pyrenoid formation?

We are currently working on characterizing the genetic knock-out of proteins identified in this manuscript. To explore whether these new proteins have a role in pyrenoid formation, we will first examine the pyrenoid morphology in these mutants by localizing common pyrenoid protein markers such as RBCS and via TEM. We will also assess CCM capacity through growth assays and O2 evolution with the assumption that significant changes in pyrenoid morphology should result in CCM disruption. Any promising candidates will further be tested *in vitro* using recombinantly expressed proteins. Experiments include membrane binding/remodeling assays and starch binding assays.

Findings can then be tested *in vivo* via domain deletions/SDMs.

47. Does pyrenoid localization = pyrenoid protein? i.e. are localizations sufficient to confirm them as pyrenoid components? Is there further validation, and how would you do it? (Not asking for more experiments)

To us a pyrenoid localization is sufficient to confirm them as a pyrenoid component, i.e. a constituent of the pyrenoid. We think this is additionally validated if there are multiple lines of support, i.e. seen in our pyrenoid proxiome or previous APMS or pyrenoid isolation and MS studies. To further validate the precise localization however, we would additionally tag known pyrenoid markers in order to establish their precise location such as periphery, matrix interface etc. However, we do not think that pyrenoid localization means they are structurally important for the pyrenoid or equates to an essential pyrenoid component. It is known that phase separated organelles often perform multiple functions, with our data also supporting additional roles for the pyrenoid.

48. How would you confirm that RBMs in your new proteins do bind Rubisco?

There are multiple ways this can be experimentally tested. SPR data of single RBMs could show binding affinity compared to the RBMs present in EPYC1. Deletion or site-directed mutagenesis of key interacting residues within the RBM could be made and localisation of the corresponding protein would confirm if the RBMs are required for correct pyrenoid targeting. Yeast two hybrid and native- PAGE gel shift assays have also been successfully used by our collaborators (McCormick lab) and ourselves, respectively.

49. Could there be additional pyrenoid components that your method did not identify? How would you find them?

We found that only 67% of the known pyrenoid proteins can be recovered in the pyrenoid proteome determined here. This is likely due to the technical limitation of proximity labeling including the (1) limited accessibility of biotin radicals across membranes, (2) Lysine availability of prey proteins. We think performing proximity labeling on additional pyrenoid components that have different sub- pyrenoid localization could further expand the pyrenoid proteome. For example, targeting TurboID to the lumen of the pyrenoid tubules could give a specific pyrenoid tubule peroxisome.

50. Question purely out of interest, does not need to be addressed: do you think that there could be proteins that operate in both the stroma and pyrenoid? i.e. that are not unique to the pyrenoid, but are required for its optimal function?

We think this is a possibility as we see multiple proteins that are continuous in both the pyrenoid matrix and stroma. However to date, all of the key structural pyrenoid components appear to be solely or highly enriched in the pyrenoid.

TPC2022-BR-01174R1 2nd Editorial decision – *acceptance pending* **April 7, 2023**

We are pleased to inform you that your paper entitled "The phase separated CO2-fixing pyrenoid proteome determined by TurboID" has been accepted for publication in The Plant Cell, pending a final editorial review by a science editor.

Final acceptance from Science Editor **May 8, 2023**
